# Polyphenols as Prebiotics in the Management of High-Fat Diet-Induced Obesity: A Systematic Review of Animal Studies

**DOI:** 10.3390/foods10020299

**Published:** 2021-02-02

**Authors:** Mohanambal Moorthy, Usha Sundralingam, Uma D. Palanisamy

**Affiliations:** 1 Jeffrey Cheah School of Medicine and Health Sciences, Monash University Malaysia, Jalan Lagoon Selatan, Bandar Sunway 47500, Selangor, Malaysia; Mohanambal.Moorthy@monash.edu; 2 Tropical Medicine and Biology Platform, School of Science, Monash University, Bandar Sunway 47500, Selangor, Malaysia; 3 School of Pharmacy, Monash University Malaysia, Jalan Lagoon Selatan, Bandar Sunway 47500, Selangor, Malaysia; Usha.Sundralingam@monash.edu

**Keywords:** polyphenols, prebiotics, gut microbiota, obesity, anti-hyperglycaemic, anti-inflammatory

## Abstract

Obesity is a disease growing at an alarming rate and numerous preclinical studies have proven the role of polyphenols in managing this disease. This systematic review explores the prebiotic effect of polyphenols in the management of obesity among animals fed on a high-fat diet. A literature search was carried out in PubMed, Scopus, CINAHL, Web of Science, and Embase databases following the PRISMA guidelines. Forty-four studies reported a significant reduction in obesity-related parameters. Most notably, 83% of the studies showed a decrease in either body weight/visceral adiposity/plasma triacylglyceride. Furthermore, 42 studies reported a significant improvement in gut microbiota (GM), significantly affecting the genera *Akkermansia*, *Bacteroides*, *Blautia*, *Roseburia*, *Bifidobacteria*, *Lactobacillus*, *Alistipes*, and *Desulfovibrio*. Polyphenols’ anti-obesity, anti-hyperglycaemic, and anti-inflammatory properties were associated with their ability to modulate GM. This review supports the notion of polyphenols as effective prebiotics in ameliorating HFD-induced metabolic derangements in animal models.

## 1. Introduction

Obesity is a public health concern as it is a risk factor for life-threatening diseases such as type II diabetes [1], cardiovascular diseases [2], certain cancers [1], kidney diseases [3], and neurodegenerative diseases [4]. In the year 2017, overweight and obesity caused over four million deaths, with more than 140 million ‘global deaths and disability-adjusted life years’ [5]. The interplay between genetic-epigenetic-environmental factors is often linked to obesity [6,7], and of late, the role of gut microbes (GM) in the development of obesity has been extensively explored [8,9,10].

The GM-obesity relationship was first highlighted by Bäckhed et al. [11] using germ-free mice, whereby conventionalisation of such mice with a normal microbiota increased body fat by 60% and elicited insulin resistance (IR) within 14 days. This finding was supported by Turnbaugh et al. [12], where the administration of microbes of obese or lean mice into germ-free mice, saw higher adiposity in the former. GM’s role was further reinforced when germ-free mice were protected against a high-fat diet (HFD)-induced obesity [13]. Collectively these studies proved the role of GM in energy harvest and obesity. Consequently, subsequent studies proposed dietary intervention for GM’s modulation, and substantial work involving probiotics, prebiotics or synbiotics in the management of obesity had emerged.

Polyphenols (PP) are secondary metabolites found abundantly in plants, fruits and nuts [14]. A growing body of research indicates that PP possess various biological activities ranging from anti-oxidant [15,16], anti-inflammatory [15], hepatoprotective [16], anti-hyperglycaemic [17,18], anti-cancer [19], neuroprotective [20,21], and cardioprotective [22]. Therefore, PP are even considered an excellent alternative to harmful food preservatives and additives [23,24,25] as well as nutraceutical agents [26]. Despite the vast health benefits, PP were shown to be poorly bioavailable and their interaction with gut microbiota was highlighted as the main factor for their bioactivities [27,28]. The PP-GM interaction in return is described to affect the intestinal ecology and influence host health [28]. Based on the International Scientific Association for Probiotics and Prebiotics consensus document, prebiotics is described as *“a substrate that is selectively utilised by host microorganisms and conferring a health benefit”* [29]. Therefore, these phytochemicals are now beginning to emerge as the new prebiotics [30].

Growing preclinical studies have confirmed the anti-obesity properties of PP to be dependent on their interaction with GM. Nevertheless, a comprehensive review is still lacking. We undertook a systematic review to investigate the effect of PP administration on obesity-related parameters, markers associated with inflammation, and gut microbiota in HFD-fed animal models. This study is essential to elucidate if PP’s effectiveness in ameliorating metabolic derangements is indeed associated with GM modulation.

## 2. Methods

The protocol of this systematic review was registered with PROSPERO (www.crd.yor.ac.uk/PROSPERO; CRD42020153321). This review’s reporting followed the Preferred Reporting Items for Systematic Reviews and Meta-Analysis (PRISMA) [31].

### 2.1. Search Strategy

MM and USL undertook a comprehensive literature search in the following databases; CINAHL, Excerpta Medica Database (Embase), Scopus, PubMed, and Web of Science. A published search term relating to PP and GM were used [30] (Table 1) (Figure S1-search strategy (PubMed)). All the acquired bibliographic records were exported to Endnote X8 (Thomson Reuters, New York, NY, USA), following which duplicates were removed. We also carried out a manual search of bibliographies of reviews and included studies to identify relevant articles.

### 2.2. Eligibility Criteria

Studies that met the following criteria were included in this review: (1) In vivo studies of any species of animal which were subjected to HFD; (2) Treatment and control animal of the same species; (3) Intervention with either pure phenolic compounds or its extracts or both; (4) Studies that measured the phenolic contents if PP rich extracts were used; (5) Studies that evaluated gut microbiota composition via next-generation sequencing methods; (6) Studies reporting on obesity-related parameters (body weight, adiposity, fasting blood glucose, fasting insulin, HOMA-IR), markers associated with inflammation (adipocytokines, endotoxins); (7) Papers published in the English language. The exclusion criteria were: (1) Combination of PP with other compounds/bacterial species. e.g., PP with fibre, PP with probiotic; (2) HFD in-combined with any diabetogens such as streptozotocin; (3) Faecal transplantation from polyphenol treated animals; (4) Fermented PP; (5) Conference abstracts; (6) Reviews/meta-analysis.

### 2.3. Data Extraction

Two reviewers, MM and USL, independently screened the titles and abstracts based on the selection criteria before evaluating the full text. Disagreements or discrepancies were discussed with a third reviewer (UDP) or resolved by consensus. The following information extracted from the full text was included in this review; the name of the first author, publication year, *animal*: Species, sex, age, and number animals per group, *intervention*: Prevention/treatment mode, percentage of fat in HFD, duration of intervention, type of PP, dose, and mode of administration, *outcomes*: Obesity-related parameters [body weight, the weight of visceral adipose tissue (VAT), the weight of subcutaneous adipose tissue (SAT), glucose, insulin, lipid profile], markers of inflammation (endotoxin, and adipocytokines), and gut microbiota modulation: Alpha-diversity, beta-diversity (based on PCoA/PCA/NMDS plots), modulation of phyla, family or genus, *Firmicutes:Bacteroidetes* ratio.

### 2.4. Quality Assessment

The methodological quality of the included studies was assessed independently by MM and USL using the SYRCLE’s risk of bias tool [32] and modified Gold Standard Publication Checklist (GSPC) [33,34]. Any disagreement or discrepancies were resolved by consensus. The SYRCLE’s checklist includes sequence generation, baseline characteristics, allocation concealment, random housing, blinding, random outcome assessment, blinding (outcome), incomplete outcome data and also other sources of bias. Each study was graded as having a low risk, high risk or unclear risk for each domain. GSPC quantitatively assesses various criteria, but only specific criteria were used in this review (Table S1) [34]. Each criterion was given ‘0’ if not addressed in the article or ‘1’ if addressed, and the total score assigned was 19.

## 3. Results

**Figure 1:**
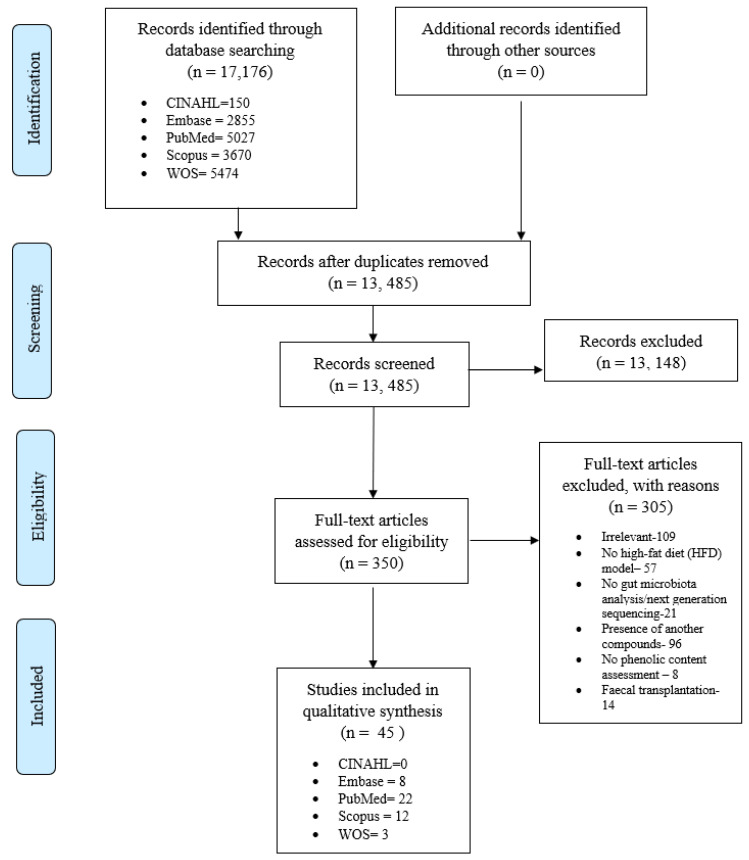
PRISMA Flow-chart.

Our database search resulted in 17,176 articles. Following duplicate removal and abstract screening, 350 full-text articles were screened. Only 45 articles that met our inclusion criteria were included in this review, as outlined in Figure 1. Table 2 summarises the characteristics of the included studies.

### 3.1. Study Characteristics

Twenty-two studies used pure single phenolic compounds, while two studies used a combination of phenolic compounds. The remaining 22 studies investigated phenolic-rich extracts. Twenty-six studies assessed the effectiveness of flavonoids, and the remaining studies investigated stilbenes or phenolic acid or a combination of flavonoids and other polyphenol classes. Most studies (*n* = 38) used mice, while only seven used rats. All the studies included in this review used male animals. The study duration ranged from 2 to 24 weeks. A majority of the studies used either 60% HFD or 45% HFD. Thirty-three studies were based on a preventive model, i.e., the intervention and HFD intake were initiated simultaneously. Ten studies used the treatment model approach; i.e., treatment began a few weeks after HFD intake. This information was not clearly stated in two studies.

### 3.2. Quality Assessment

Risk of bias of included studies is summarised in Figure S2. Sequence generation, allocation of concealment, random outcome assessment and blinding (detection) were unclear in all the studies. Baseline characteristics were reported in 98% of the studies, and incomplete outcome data was low in 96% of the studies. The animals were randomly housed in 73% of the studies while this was not carried out in 27% of the studies. The study quality was also assessed using the GSP checklist, which provided a quantitative assessment of each study. As depicted in Figure S3, five studies scored above 13 with the highest score being 14, while 40 studies scored between 7–12 with the lowest score being 9. Most studies did not address sample-size calculation, concealment of allocation, the animal’s weight, time of intervention, method and time of sampling, number, and reason of excluded animals. The detailed GSPC scores are presented in Table S1.

### 3.3. Effect of Polyphenols on Food/Energy Intake, Obesity-Related Parameters, and Markers-Associated with Inflammation

The results are presented as significantly high (SH), significantly low (SL), or as not significant (NS) which refers to either a parameter being significantly increased, decreased or not-significant compared to HFD group. Studies that reported more than a compound or tested various dosages are presented as separate studies.

#### 3.3.1. Effect of Polyphenols on Energy/Food Intake and Bodyweight

Thirty-two studies reported changes in energy/food intake, among which three studies that used pure phenolic compounds (PPC) [35,40,42,43] and two [68,78] studies that investigated phenolic extracts (PE) reported a significant drop in energy/food intake compared to HFD. Significantly higher food consumption was recorded in three studies [2-PPC [41,50], 1-PE [73], while 24 studies reported non-significant difference in energy/food intake [45,46,47,51,52,57,59,60,62,63,66,74,75,80] (Figure S4). As for bodyweight, thirty studies (67%) recorded a significant drop post-treatment, of which 16 with PPC [35,36,37,38,39,40,41,42,43,44,45,50,52,53,54,56,57], and 15 with PE [60,61,63,65,66,68,71,72,73,74,75,76,77,78,79,81]. Non-significant observations were made in 19 studies [11-PPC [37,38,44,46,47,48,49,51,55,58], 9-PE [59,60,62,64,65,67,69,70,80]] (Figure S4).

#### 3.3.2. Effect of Polyphenols on Adiposity

The adiposity in animal models was presented as the weight of visceral adipose tissue (VAT), subcutaneous adipose tissue (SAT) or total body fat (TBF). The VAT was significantly reduced in 24 studies [PPC-14 [37,38,39,41,42,43,44,46,48,50,51,52,53,54,57], PE-[61,63,65,68,71,72,73,77,78,81]], and 11 studies reported non-significance [40,44,45,49,65,67,69,70,76,80,81]. As for the SAT, seven studies showed a significant reduction [4-PPC [35,37,40,42,43], 3-PE [61,62,73]], while 10 studies showed non-significant findings [36,38,39,44,45,46,48,49,51,53,54,55,56,57,58,59,63,65,66,71,74,75,77,79,80,81]. The TBF was significantly lowered in two studies that investigated PPC [60,81] while three studies [60,62,64] indicated non-significant changes (Figure S5).

#### 3.3.3. Effect of Polyphenols on Lipid Profile

Total cholesterol was significantly lowered in 11 studies following the administration of PPC [37,38,41,42,43,46,50,52,54,56,57,58] and eight studies for PE [63,68,71,73,74,75,76,79,81]. One study reported a significant increase with PPC [45]. Thirteen studies reported non-significant findings [35,36,38,46,49,55,56,66,67,71,72,79,81]. As for TAG, PPC significantly lowered the index in 12 studies [35,37,39,41,42,43,44,50,52,54,55,57,58], and PE in 11 studies [60,61,63,65,68,70,72,73,76,79,81]. Nine studies that used PPC significantly lowered LDL [36,38,44,46,52,54,56,57,58] level and eight studies showed a similar result with PE [63,65,66,71,76,77,79,81]. As for the HDL level, four studies recorded a significant reduction [3-PPC [38,46,56], 1-PE [63]], and five studies a significant increment [5-PPC [44,45,52,54,55], 2-PE [66,81]] (Figure S6).

#### 3.3.4. Effect of Polyphenols on Glucose Homeostasis

The fasting glucose (FG) was reported in 24 studies, from which nine studies reported a significant reduction following administration of PPC [37,38,39,41,42,44,48,55,57] and eight for PE [63,64,65,72,74,75,76,77,81]. Twelve studies reported non-significant changes in FG [35,38,50,55,62,64,65,66,68,70,73,77]. The oral/intraperitoneal glucose tolerance test (GTT) was evaluated in 21 studies, and among these, five PPC [41,42,43,47,53,57] and nine PE [59,60,61,62,64,67,72,76,77] showed significant improvement in glucose tolerance, and 12 studies reported non-significant changes [48,51,55,59,60,62,64,67,68,69,70,80]. Fasting insulin (FI) was significantly lowered in four studies with the use of PPC [39,42,43,48,54] and six studies with PE [65,66,68,70,72,77]. Non-significant observations were recorded in nine studies [55,57,62,65,66,67,70,73,80]. HOMA-IR was significantly lowered in 13 studies [4-PPC [39,48,55,57], 9-PE [60,65,66,67,68,72,73,74,75,77]], and five studies recorded non-significant changes [55,60,65,66,67] (Figure S7).

#### 3.3.5. Effect of Polyphenols on Adipocytokines, CRP, and LPS/LBP

Thirteen 13 studies investigated TNFα level in serum/plasma, from which 12 studies recorded a significant reduction [36,37,41,44,46,51,54,61,66,71,74,75,79] and two studies recorded non-significant changes [37,44]. As for IL-6, 11 studies showed a significant reduction [36,37,39,46,51,54,61,66,71,74,79], while two studies reported non-significant changes [37,79]. MCP-1 was significantly lowered in four studies [51,54,60,74,75], and two studies [60,74,75] recorded non-significant observations. Meanwhile, leptin was significantly reduced in three studies [36,42,43,61], and two studies recorded non-significant findings [54,67]. Adiponectin was only evaluated in two studies, of which one showed a significant increment [54], and another recorded a non-significance [42,43]. The CRP level was significantly lowered in two studies [46,71]. The LPS/LPB was significantly reduced in 12 studies that investigated PPC [35,36,37,39,41,42,43,44,46,48,50,56,57] and 11 studies that investigated PE [60,61,63,66,68,70,71,73,74,75,79,80]. Six studies recorded non-significant changes in LPS/LBP concentrations [37,60,62,66,70,79] (Figure S8).

#### 3.3.6. The Overall Effect of Polyphenols on Obesity-Related Parameters and Inflammation

**Figure 2:**
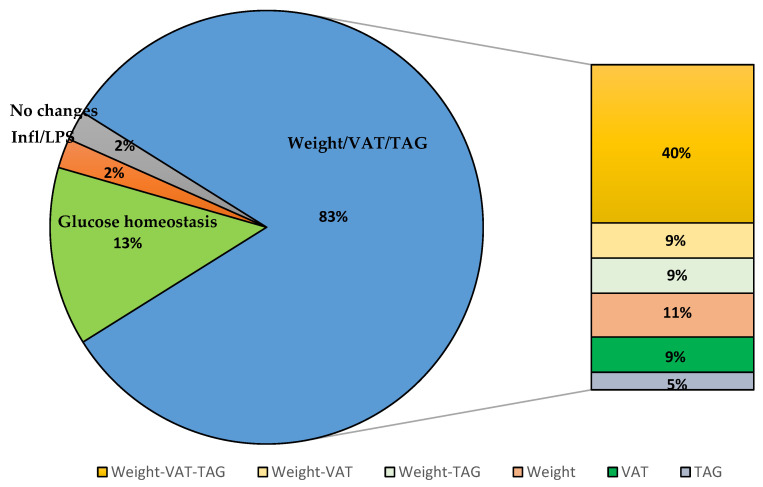
Overall effect of the phenolic intervention on metabolic parameters. VAT-visceral adipose tissue, TAG-triacylglyceride, Inf-inflammatory markers, LPS-lipopolysaccharide.

Figure 2 summarises the overall impact of the phenolic intervention on various obesity-related parameters and inflammation. The most profound effects (83%) were seen on either body weight or visceral adiposity or TAG or all three [35,36,37,38,39,40,41,42,43,44,45,46,47,48,50,51,52,53,54,56,57,58,60,61,63,65,66,68,70,71,72,73,74,75,76,77,78,79,81]. While 13% of the study saw improvement only in glucose parameters [55,59,62,64,67,69], and 2% (one study) of the study reported improvement in inflammatory markers and LPS [80]. The remaining 2% (one study) of the study did not significantly improve the metabolic parameters measured [49].

### 3.4. Effect of Polyphenols on Gut Microbiota

We report the changes in GM as alpha diversity, beta-diversity, *Firmicutes: Bacteroidetes* ratio (F:B ratio), overall changes in phyla, family/genus and frequently modulated genera.

#### 3.4.1. Alpha and Beta-Diversity

The included studies’ alpha-diversity were reported as ACE, Chao1, OTU, Shannon, Simpson, Inverse Simpson or PD. The alpha-diversity was significantly increased in 10 studies [4-PPC [41,54,55,57], 6-PE [60,61,63,71,73,76]], while six studies recorded a significant decrease [4-PPC [44,46,49,53], 2-PE [65,71]]. Non-significant observations were recorded in 17 studies [10-PPC [35,40,41,44,45,46,48,49,51,52], 7-PE [59,60,65,71,72,74,75,81]] (Table S2). The beta-diversity was based on the PCoA/PCA/NMDS plots. Among the studies which tested PPC, 12 studies showed improvement in beta-diversity [36,37,41,42,43,45,46,47,49,51,53,54,55], and 14 studies for PE [59,61,62,63,65,68,69,70,71,72,73,76,77,78], i.e., there were formations of clusters near normal-diet fed rats or formations of distinct cluster away from normal or high-fat-fed animals. Nine studies showed no improvement [PPC [39,40,44,46,48,56,57], PE [60,70]], i.e., there were formations of clusters with HFD/some overlapping with HFD (Figure S9).

#### 3.4.2. Modulation of *Firmicutes:Bacteroidetes* Ratio (F:B Ratio), Phyla, and Family/Genus

**Figure 3:**
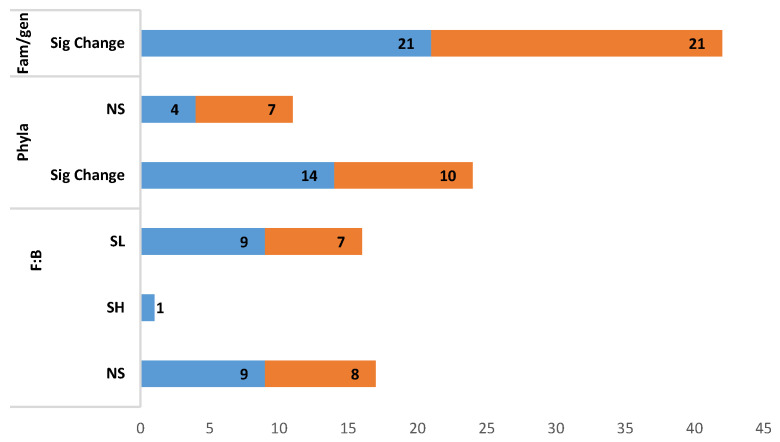
Effect of polyphenols on Firmicutes:Bacteroidetes ratio, phyla, and family/genus. SL-Significantly Low, SH-Significantly High, Sig change-significant changes, NS-Not Significant, Fam-family, gen-genus. Significance/non-significance compared to HFD. Studies that tested more than one compound/dose: [45,54,57,58,62,63].

As presented in Figure 3, F:B ratio was significantly lowered in nine studies that tested PPC [37,39,42,43,45,47,49,55,56,57] and seven studies for PE [61,69,71,72,77,79,81]. Non-significance was recorded in 17 studies [9-PPC [36,37,44,46,48,52,54,55,58], 8-PE [59,62,64,71,73,74,75,77,79]]. At the phyla level, significant changes were reported in 24 studies [36,37,39,40,41,45,46,49,50,51,54,55,56,57,62,66,68,70,71,76,77,78,80,81], while 42 studies showed a significant modulation of either family/genus [36,37,39,40,41,42,43,44,45,46,47,48,49,50,51,52,53,54,55,56,57,58,59,60,61,62,63,64,65,66,67,68,69,70,71,72,73,76,77,78,79,80,81,82,83]. Eleven studies did not observe any significance in phyla [48,52,53,58,59,62,64,65,67,72,73]. Three studies did not perform any statistical analysis [35,38,74,75].

#### 3.4.3. Frequently Modulated Gut Microbes

**Figure 4:**
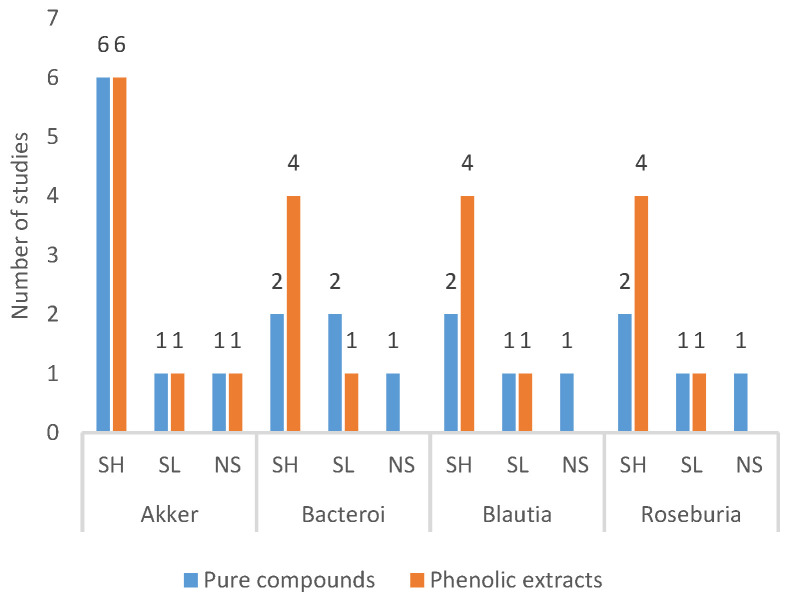

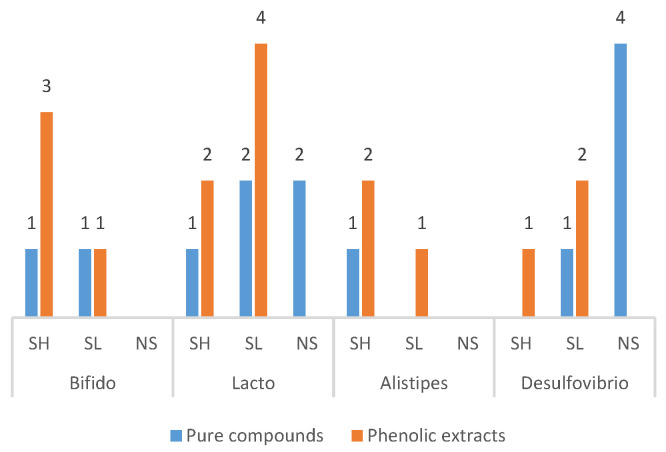
Frequently modulated gut microbes. Akker-Akkermansia, Bacteroi-Bacteroides, Bifi-do-Bifidobacterium, Lacto-Lactobacillus, SL-Significantly Low, SH-Significantly High. NS-Not Sig-nificant. Significance/non-significance compared to HFD.

Figure 4 explains the most frequently modulated gut microbes following the phenolic interventions. The most notable improvement was observed for *Akkermansia*, whereby 12 studies reported a significant increase [six-PPC [37,41,42,43,46,49,57], six-PE [61,66,68,70,73,77]], while two studies observed a significant reduction [47,76]. *Bacteroides* was significantly increased in six studies [37,47,59,66,71,78], and three reported a significant reduction [51,58,76]. *Blautia* and *Roseburia* were upregulated in six studies [51,55,62,63,64,65,66,67] and downregulated in two [56,58,78,79]. *Bifidobacterium* was significantly high in four studies [one-PPC [35], three-PE [61,66,73]] and lowered in two studies [37,70], *Lactobacillus* was significantly high in three studies [48,76,79] and significantly low in six studies [56,57,68,70,71,78]. *Alistipes* was significantly increased in three studies [57,66,71] and significantly reduced in one [76]. *Desulfovibrio* was significantly increased in one study [64] and decreased in three studies [39,61,67].

## 4. Discussion

Polyphenols are emerging as prebiotics due to their ability to improve intestinal dysbiosis and health parameters [29,30]. Numerous preclinical studies have investigated the role of polyphenols in improving various health conditions in animal models. This comprehensive review was carried to obtain a complete and exhaustive summary of the preclinical studies investigating the effect polyphenols in ameliorating HFD-induced obesity.

In general, 98% of the studies reviewed indicated significant improvement in the metabolic derangements inflicted by HFD intake after consuming pure phenolics compounds or its extracts. These changes were related to the modulation of GM at the genus level. Our analysis also indicates that all 23 studies that tested pure PP and 21 out of 22 studies used extracts observed improvement in metabolic parameters and gut microbiota profiles. Thus, we can conclude that both pure PP and extracts were equally effective as prebiotics. However, this review’s findings will need to be interpreted with some caution as the quality score for the majority of studies was below 12. This was because a few essential criteria such as sequence generation, allocation of concealment, random outcome assessment, blinding (detection), and sample size calculation were not addressed in most studies.

### 4.1. Effect of Polyphenols on Obesity-Associated Parameters, Adipocytokines, and LPS/LBP

It is evident that phenolic intervention significantly reduced one or more metabolic parameters such as weight gain, VAT, FBG, FI, GTT, HOMA-IR, TC, TAG, LDL, LPS, TNFα and IL-6 in 98% of the studies included in this review.

The most prominent observation in this review was the reduction in body weight/VAT/TAG reported in 83% of the studies. Among these, 40% of studies showed a decrease in all three parameters, i.e., body weight, VAT and TAG. Therefore, it can be assumed that the reduction in body weight is probably related to reduced VAT and TAG. The mechanisms highlighted for TAG improvement in some of these studies include downregulation of *Agpat2* [60], reduction in taurine-conjugated bile acids causing activation of nuclear farnesoid X receptor (FXR) in the liver, consequently, suppressing lipogenic pathway in the liver [61], inhibition of hepatic adipogenic genes such as Fas, PPAR*γ*, and SREBP-1c [71,72], and upregulation of fasting-induced adipose factor (FIAF) expression in the intestine, hence, suppressing TAG deposition [81]. Our observation indicates that the mode of action varies between different PP.

### 4.2. Effect of Polyphenols on Glucose Homeostasis

We also observed that studies reporting a reduction in weight, VAT, and or TAG revealed a significant improvement in glucose homeostasis (either FBG, GTT, FI or HOMA-IR), and its inflammatory state. However, whether the improvement in glucose homeostasis is a cause or effect of a decreased inflammatory state is unclear. Although many studies have hypothesised that low-grade systemic inflammation in white adipose tissue disrupts glucose homeostasis, a clear mechanism of HFD-induced hyperglycaemia or IR remains elusive [84,85,86]. Few other studies mention that IR precedes WAT inflammation. Shimobayashi et al. [87] reported that the accumulation of macrophages in WAT preceded IR in HFD and mTORC2-knockout animal models. They further stated that IR in WAT induced MCP-1 production responsible for the recruitment of monocytes and activation of macrophages in WAT. Lee et al. [88], on the other hand, observed IR and glucose intolerance as early as day 3 following HFD intake, and this was associated with lipid overload in the adipose tissue. Chronic inflammation was identified as a critical mechanism for IR once obesity is established. Apart from this, ellagic acid and its metabolites were demonstrated to mitigate IRs by improving mitochondrial function by activating 5′-AMP-activated protein kinase [89,90,91]. Some PPs such as geraniin, corilagin, and ellagic acid are also shown to exhibit insulin-like activity, causing 2-NBDG uptake in the absence of insulin by preadipocytes [92].

Few PPs only improved glucose intolerance/IR without affecting weight/VAT/TAG. The exact mechanisms for this were not well elaborated in the papers included in this review. Nevertheless, the mechanism of actions was discussed extensively in a few reviews [14,93,94,95]. Some of which include; inhibition of salivary and pancreatic α-amylase and α-glucosidase activities. These enzymes are responsible for metabolising carbohydrate and its inhibition of reduced carbohydrate metabolism and therefore, glycaemic spike [14,94]. This effect has been reported with punicalagin [96], ferulic acid, and isoferulic acid (α-glucosidase inhibitors) [97]. Certain polyphenols were also shown to inhibit glucose transporters in the gut, therefore reducing glucose uptake. For instance, quercetin, chamomile and green tea were shown to inhibit glucose uptake in the intestine by inhibiting GLUT2, and GLUT5 [98,99]. Some polyphenols such as isovanillic acid 3-O-sulfate (metabolites of cyanidin 3-O-glucoside) [100] and epicatechin [101] have been shown to increase glucose intake by upregulating GLUT4 expression in tissues.

### 4.3. Effect of Polyphenols on Gut Microbiota

Approximately 93% of the studies included in this review reported significant changes in one or more parameters of GM modulation. It is unclear whether the remaining 7% of the studies (three studies) performed any statistical analysis to prove GM modulation and only reported their findings in relative abundance changes. Nevertheless, these studies did report positive changes with phenolic interventions.

The alpha-diversity was reported in 29 studies (64%); among these nine studies reported a significant increase in this diversity. Beta-diversity was presented as a PCoA plot in most studies. Among the 35 studies that reported this diversity, 26 plots showed distinct cluster/clustering closer to normal diet groups. The phenolic intervention did not improve alpha-diversity in most studies, which could be due to the shorter study duration [102]. Furthermore, diet-diversity was previously observed to affect beta and not alpha-diversity [103].

F:B ratio was reported in 28 studies, with 17 studies indicating a significant reduction of the ratio. This raises the question of phyla level relevancy often reported in obesity studies. Higher *firmicutes* and lower *bacteroidetes* (high F:B ratio) are often associated with obesity [104,105,106]. On the contrary, few studies showed a low F:B ratio [107,108,109]. A few meta-analyses have also shown a lack of differences in F:B ratio between obese and lean individuals [110,111,112]. It was suggested that the F:B ratio may not be a robust indicator of dysbiosis in obesity [113]. It was further highlighted that the disparities that exist between studies could be related to methodological differences such as sample collection, DNA extraction, hypervariable regions of interest, sequencing methods and bioinformatics data analysis software (QIIME, Mothur) [113].

Nevertheless, polyphenols significantly improved relative abundance of GM at the family/genus level in 42 studies. A few genera were consistently observed to be modulated irrespective of the type of intervention. The commonly improved genera include *Akkermansia*, *Bacteroides*, *Blautia*, and *Roseburia*, and to a lesser extent, *Bifidobacteria*, *Lactobacillus*, and *Alistipes*. *Desulfovibrio,* on the other hand, was reduced in three studies. The genus *Akkermansia* was significantly increased with the intake of vanillin, anthocyanin, kudingcha dicaffeoylquinic acids, EGCG, quercetin-resveratrol, total flavonoid, blueberry extract, tea polyphenols, cranberry extracts, cloudberry extract, alpine bearberry extract, green tea extract, and olive leaf extract. *Akkermansia muciniphila (A. muciniphila)*, is a gram-negative bacterium belonging to the *Verrucomicrobia* phylum. This bacterium produces mucin degrading enzymes causing fermentation of mucin to acetic, propionic acid, and sulphate [114]. A preclinical study reported that *A. muciniphila* reduced plasma TAG, improved fatty liver, and gut dysbiosis [115]. In a randomised human trial, administration of *A. muciniphila* for 30 days improved plasma insulin, HOMA-IR, Dipeptidyl-peptidase 4 (DPP4) (inflammation) and LPS concentration in overweight/obese individuals [116]. Administration of procyanidins, resveratrol, Rubus occidentalis, tea polyphenols, kudingcha tea, fuzhuan brick tea, green tea polyphenol, and black tea polyphenol improved *Bacteroides.* Some *Bacteroides* strains were categorized as next-generation probiotics [117], including *B. uniformis, B. acidifaciens,* and *B. dorei.* These strains were shown to ameliorate HFD-induced metabolic derangements [117].

In contrast, some strains of *B. fragilis* were identified as a risk factor for the development of colon cancer [118]. It can therefore be concluded that the health benefit of the genus is strain specific. *Blautia* was improved with grape seed proanthocyanidin extract, resveratrol, Rutgers scarlet lettuce, green tea, oolong tea and black tea, rapeseed oil with sinapine, tea polyphenols. This genus is a butyric producer, reported to ameliorate intestinal inflammation, IR [119], and exhibited an inverse association with visceral adiposity [120]. Therefore, it may play a crucial role in the management of obesity and obesity-related diseases. *Roseburia* was improved with grape seed proanthocyanidin extract, a combination of resveratrol and sinapic acid, Rutgers scarlet lettuce, grape seed extract, tea polyphenols, grape pomace extract. *Roseburia* also a butyrate producer and this genus has been implicated in the control of intestinal inflammation and was shown to be reduced in patients with type II diabetes [121]. The conventional probiotics such as *Bifidobacterium* and *Lactobacillus* were improved in fewer studies. Administration of kudingcha dicaffeoylquinic acids, blueberry extract, and tea polyphenols improved *Bifidobacterium*, while *Lactobacillus* was improved with the intake of hydroxytyrosol, pandanus tectorius fruit extract, pomegranate polyphenols. Some of the health benefits of *Bifidobacterium* include improvement in celiac disease with the administration of *B. infantis* strain NLS [122], irritable bowel syndrome with *B. lactis* DN-173010 [123]. Conversely, *Lactobacillus* was significantly improved in three studies, while six studies showed a significant reduction.Since only the changes up to genus level were reported in all the studies, it is uncertain the type of species/strains affected following phenolic interventions. Literature suggests that the health benefits of *Lactobacillus* are species/strain-dependent. For instance, weight loss was reported with *L. gasseri* [124], and *L. reuteri* JBD301 [125]. Whilst, weight gain was reported with *L. acidophilus* [126]. The abundance of *Alistipes* improved with the administration of total flavonoids of quzhou, tea polyphenols, kudingcha and fuzhuan brick tea. Similar to *Lactobacillus*, the health attributes of this genus are species/strain specific. Among patients with liver cirrhosis, lower *A. shahii* and *A. putredinis* levels were observed compared to healthy controls [127], while *A. finegoldii* was shown to promote colorectal cancer [128]. *Desulfovibrio* is known to produce a cytotoxic compound; hydrogen sulfide. This bacterium was observed to increase in patients with ulcerative colitis [129].

### 4.4. Is LPS the Only Linking Factor between Metabolic Derangements and Gut Dysbiosis?

Consumption of HFD was proven to increase fat deposition, elicit IR, and inflammation in adipose tissue, liver and skeletal muscle [130,131]. LPS was discovered as one of the linking factors between HFD intake and metabolic derangements. The administration of LPS into lean mice on normal-diet caused similar changes as the HFD-fed mice [130]. Therefore, we explored the relationship between GM, LPS, and metabolic parameters. Among the 23 studies that reported a significant reduction in LPS/LBP, 20 stated significant modulation in GM and metabolic derangements following PP administration. Two studies [35,74] reported significant improvement in metabolic parameters and LPS concentration; however, they did not indicate GM’s statistical significance. Thus, the relationship between GM modulation and LPS is unclear for these two studies. Interestingly, Cheng et al., 2016 [62], reported non-significance for LPS even though GM modulation and significant improvement in OGTT were reported with Rutgers scarlet lettuce. This suggests that other factors may also play a role in the amelioration of metabolic derangements such as bile acids, and short-chain fatty acids (SCFA). Gut microbiota composition has been shown to alter the type of secondary bile acids, and consequently influence glucose homeostasis [132]. It has also been shown to improve SCFA production; for instance, the administration of butyrate was shown to improve rodent’s insulin sensitivity [133]. Considering the studies included in this review, we conclude that PP’s administration improved GM composition and consequently, the metabolic parameters. This was achieved partly by reducing LPS concentration; however, the involvement of other mechanisms cannot be discounted.

## 5. Limitations

In general, PP interventions significantly improved HFD-induced metabolic derangements in 98% of the animal studies and these changes were shown to be related to the GM modulation. Nevertheless, there are some limitations to this review, which will be discussed here.

### 5.1. Experimental Animals and Their Environment

A variety of experimental animal species (C57BL/6J, C57BL/6N, C57BL/6 wild type Sprague Dawley rats, Wistar rats) of varying ages were used. These experimental animals were exposed to different types of a high-fat diet, water source, bedding materials, and were either housed alone or in groups.

### 5.2. Types of Intervention

Different types of phenolic compounds were tested, either in its pure form or as an extract at varying doses. These compounds were also administered via various modes (gavage/within diet or drinking water).

### 5.3. Methodology to Assess GM

GM modulation may have been affected due to variations in the methodology employed between studies, including the use of faecal samples or intestinal contents, faecal sampling time, and the difference in the hypervariable regions, sequencing methods, and bioinformatics tools utilised.

### 5.4. Outcomes Reported

The parameters reported for metabolic derangements and GM modulation differed, i.e., not all studies reported the same parameters. These variations greatly influenced the reported outcomes, thus posing a challenge in summarising polyphenols’ prebiotic effect in preclinical studies.

## 6. Conclusions

Polyphenols appear to be a promising phytochemical in the management of diet-induced obesity. This review, havedemonstrated that the administration of polyphenols to obese animals improved metabolic derangements, particularly, weight gain, visceral adiposity, plasma TAG, and glucose homeostasis inflicted by HFD intake. These improvements were established to be the result of improved gut dysbiosis as presented in all the studies. The findings can form the basis for the development of randomised clinical trials required to conclusively place polyphenols as prebiotics. Moreover, Nonetheless, discrepancies in study designs, metabolic markers measured, and the methodology of gut microbiota analysis in preclinical studies need to be streamlined to make a more robust conclusion.

## Figures and Tables

**Table 1:** Search terms.

Polyphenol	Gut Microbiota
Dietary polyphenol Polyphenol Flavonoid Fruit Vegetable Plant extract Herbal drug Medicinal plant Antioxidant Anthocyanin Chalcones Catechin Flavanone Proanthocyanidin Ellagitannin Functional food Green tea Puerh tea Cocoa Chocolate Myo-inositol Soy isoflavone Blueberries Berries Grape Quercetin Citrus Cinnamon Red wine Resveratrol Natural s-equol	Microbiota Gut microbiota Colonic microbiota Gastrointestinal microbiota Intestinal microbiota Gut organism Microbial consortia Gut bacterium Gut flora Gastrointestinal flora Intestinal flora

**Table 2:** Characteristics of reviewed animal studies.

No.	Author, Yr, [Reference]	Species, Sex, Age (w)/ Weight (g)	Number of Animals, *n*/ Groups (Grp)	Prevention (*p*)/Treatment (T)	Duration of Intervention (d/wks/mths)	HFD (%Fat)	Dosage	MOA	Sample/Method/ Hypervariable Region/GM Composition	Energy/Food Intake	Weight	VAT/ SAT	Glucose (FG/GTT/ITT)	Hormones	HOMA-IR	Adipocytokine	Lipid Profile	Endotoxins
	**PURE COMPOUNDS-MICE**																	
1	López et al., 2018 [35]	C57BL6 mice, M, 9 w	8/grp ND HFD + genistein(G)	*p*	24 w	NA	Genistein −0.2%	Within diet	Feces, 16s rRNA, V3-V4 regions, Illumina MiSeq: Alpha diversity−high Phyla: Firmicutes-H Bacteroidetes−L Verrucomicrobia-H Genera: Bacteroides-L Prevotella and Akkermansia-H Species: Prevotella copri, Prevotella stercorea, Akkermansia muciniphila-H Bacteroides acidifaciens and Bacteroides uniformis-L	SL	SL	SAT:SL	FG−NS IpGTT(AUC)−SL				TC-NS TAG-SL LDL-NS	LPS-SL
2	X. Guo et al., 2018 [36]	C57BL/6J mice, M, 4 w	12/grp ND HFD HFD + R HFD + R + Inulin * only results for rutin reported in this review	*p*	20 w	60%	Rutin (R)-6.4 mg/g diet	Within diet	small intestine content, 16s rRNA, V4 region, Illumina HiSeq: Beta-diveristy (PCoA)-cluster near ND Phyla: *Firmicutes*-SL *Bacteroidetes*-NS *Deferribacteres*-NS *Actinobacteria*-NS *Proteobacteria*-SH F:B ratio-NS Families: *Rikenellaceae*-NS *Porphyromonadaceae*-NS *Bacteroidaceae*-SH *Bacteroidales*_S24-7 group-SH *Deferribacteraceae*-NS *Erysipelotrichaceae*-SH *Ruminococcaceae*-NS *Desulfovibrionaceae*-SH *Helicobacteraceae*-NS *Lachnospiraceae*-SL LDA > 3 (genus): *Desulfovibrio*	NS	SL					Leptin-SL IL-6-SL TNFa-SL IFNy-SL IL-2-SL IL4-SL	TC-NS TAG-NS	LPS-SL
3	Masumoto et al., 2016 [37]	C57BL/6Jmice, M, 9 w	10/grp ND HFHS HFHS + OP HFHS + PP	NA	20 w	NA	Oligomeric procyanidins (OP)-NA Polymeric procyanidins (PPs)-0.5%	NA	Cecal content, 16S rRNA, V3–V4 regions, Illumina MiSeq: Beta-diversity (PCoA)-Distinct cluster for OP and PP Phyla: *Firmicutes*-SL (PP) *Bacteroidetes*-NS *Verrucomicrobia*-NS *Proteobacteria*-NS *Tenericutes*-NS *Deferribacteres*-NS *Actinobacteria*-NS Significance observed with PP only for the following genera: *Bifidobacterium*-SL *Adlercreutzia*-SH *Bacteroides*-SH *Rikenellaceae*-SH *S24-7-SH Lachnospiraceae*-SL *Roseburia*-SH *Ruminococcus*-SL *Peptococcaceae*-SL *rc4-4-SH Ruminococcus*-SL *Anaerovorax*-SH *Anaeroplasma*-SH *Akkermansia*-SH	OP, PP-NS	OP-NS, PP-SL	VAT: OP, PP-SLSAT: OP, PP-SL	OP, PP-SL			TNFa: PP-SL IL-6:PP-SL	TC: OP, PP-SLTAG:OP, PP-SL	LPS:PP-SL
4	Tung et al., 2016 [38]	C57BL/6 mice, M, 5 w	8/grp ND HFD HFD + 0.1%R HF+ 0.1% Pic (LP)HFD+ 0.25% Pic(HP)	*p*	18 w	45%	0.1% Resveratrol (R), 0.1% Piceatannol (Pic), 0.25% Pic(1 kg of HFD contained 1 or 2.5 g Pic powde)	Withi *n* diet	Feces, 16S rRNA, V4 region, Illumina MiSeq:* No statistics Phyla: *Firmicutes*: LP, HP-H *Bacteroidetes*: LP, HP-L Genera:*Bifidobacterium*: LP, HP-L *Lactobacillus*: LP, HP-H*Pedobacter*: LP-L, HP-H*Blautia*:LP-H, HP-L *Dysgonomonas*: LP, HP-L	R, LP, HP-NS	LP, HP-SL	Perigonadal: R, LP, HP-SL RP: R, LP, HP-SL Mesen: R, LP, HP-NS	FG:LP, HP-SL				TC: LP, HP-SL TAG:LP, HP, R-NS LDL:HP-SL HDL: LP, HP-SL	
5	Porras et al., 2017 [39]	C57BL/6J mice, M, 7 w	10/grp:NDND + QHFDHFD + Q	*p*	16w	60%	Quercetin (Q)-0.05% (*w/w*) aglycone quercetin	Within diet	Cecal content, 16 srRNA, V3–V4regions, Illumina MiSeq: Beta-diversity (PCoA)-cluster with HFD Phyla: *Firmicutes*-NS *Bacteroidetes*-NS *Proteobacteria*-SL F:B ratio-SL Genera: *Desulfovibrio*-SL *Helicobacter*-SL *Flavobacterium*-SH*Allobaculum*-SH *Sutturella*-SH *Blautia, Akkermansia, Oscillospira, Parabacteroides, Alkaliphilus, Lactobacillus*-NS	NS	SL	Epid-SL	FG-SL	FI-SL	SL	IL-6-SL	Plasma:TAG-SLLiver:TAG, FFA-SL	LPS-SL
6	Brandt et al., 2018 [40]	C57BL/6N with loxP insertions in the Ppargc1a gene, M, 8–10 w	*n* = NANDHFDHFD + R HFD + exercise (Ex) * only results for resveratrol reported in this review	*p*	16 w	60%	Resveratrol (R)-4 g/kg HFD	Within diet	Colon sample, 16s rRNA, V3–V4 region, GS FLX titanium pyrosequencing: Alpha diversity (Shannon index)-NSBeta-diversity (PCoA)-distinct centroids were observed for each treatment, SL Phyla affected by R: *Proteobacteria, Verrucomicrobia* Genera: *Allobaculum*-SH*Alistipes*-NS *Dorea*-NS	SL	SL	VAT: NSSAT: NS				Serum Amyloid A-NS		
7	J. Guo et al., 2018 [41]	C57BL/6J mice, M, 3 w	7–8/grp ND HFDHFD + V	*p*	14 w	60%	0.1% vanillin	Within diet	Pooled content of colon, rectum and cecum, 16r rRNA, Illumina HiSeq: Ace index, Chao1, OTU (richness)-SHShannon and Simpson (homogeneity)-NSBeta-diversity(PCoA)-similar to NDPhyla: *Firmicutes*-L *Proteobacteria*-L *Verrucomicrobia*-SH *Actinobacteria*-H Genera: (LDA > 3) *Akkermansia, Romboutsia, Peptoclostridium*	SH	SL	Epid-SL Inguinal-SL	FG-SL OGTT(AUC)-SL ITT(AUC)-SL			TNFa-SL IL-6-SL	TC-SL TAG-SL LDL-SL HDL-NS	LPS-SL
8	Cremonini et al., 2019 [42], Daveri et al., 2018 [43]	C57BL/6J mice, M, (20–25 g)	10/grp NDND + AC HFDHFD + AC	*p*	14 w	60%	Anthocyanins(AC)-40 mg/kg	Within diet	Cecal content, 16s rRNA, V4 region, Illumina MiSeq: Beta-diversity(NMDS)-closer to ND F:B ratio-SL *Akkermansia*-SH	SL	SL	VAT: SL SAT: SL	FG-SL OGTT(AUC)-SL ITT(AUC)-SL	FI-SL GIP, GLP-1-SL GLP-2-SH		Leptin-SL Adiponection-NS	Plasma: TC-SL TAG-SL Liver:TAG-SL	Endotoxin-SL FITC-dextran-SL
9	Campbell et al., 2019 [44]	C57BL/6J mice, M, 4 w	12/grp ND HFD HFD + L HFD + M HFD + H	T	12 w	35%	HFD + L − 50 mg/kg/day HFD + M − 75 mg/kg/day HFD + H − 100 mg/kg/day Resveratrol were dissolved in 0.4 mL of absolute ethanol and added to 100 mL of drinking water daily	Drinking water	Cecal content, 16s rRNA, V4 region, Illumina MiSeq: Alpha diversity (Chao1)-MD, HD-SHPCoA-RSV improved the GM shift caused by HFD but not completely Family:* Desulfovibrionaceae*-LD, MD, HD-NS *Prevotellaceae*-LD, MD, HD-NS *Verrucomicrobiaceae*-LD, MD, HD-NS *Deferribacteraceae*-MD-SH, LD, HD-NS	LD, MD, HD-NS	LD-NS MD-SL HD-SL	Epid, perinephric, mesen:LD-NS MD-SL HD-SL	FG: LD, MD, HD-SL			IL-1: MD, HD-SLIL-10: LD, MD, HD-SL TNFa: MD, HD-SL	TAG: LD, MD, HD-SL HDL:HD-SH LDL:LD, MD, HD-SL	LPS:LD, MD, HD-SL LBP: LD, MD HD-SL
10	Zheng et al., 2018 [45]	C57BL/6J mice, M, 3 w	6/grp: LFHFD HFD + *p*	T	12	60%	Procyanidin(*p*) 100 mg/kg	Oral gavage	Feces, 16s rRNA, V3–V4 regions, Illumina MiSeq:Alpha diversity:Simpson: NSBeta diversity (Bray curtis): SH Phyla: *Firmicutes*: NS *Bacteroidetes*: SH F:B ratio-SL Genera: *Rikenellaceae RC9* gut group-H *Blautia*-H *Anaerotruncus colihominis*-H *Helicobacter hepaticus*-H *Rikenella*-L *Lachnospiraceae_FCS020_group*-L *Clostridiales_bacterium_CIEAF_020*-L *Lachnospiraceae_UCG-006*-L *Peptococcus*-L *Ruminococcaceae*-L *[Eubacterium] _coprostanoligenes_group*-L *Ruminiclostridium*-L *Ruminiclostridium*_5-L *Ruminococcaceae_UCG-004*-L *Ruminococcaceae_UCG-014*-L *Desulfovibrio*-L		SL	VAT: NS					TC-SH TAG-NS HDL-SH LDL-NS	
11	Xie et al., 2018 [46]	C57BL/6 mice, M, 6 w	8/grp ND ND + LD HFD HFD + LD HFD + HD	*p*	9 w	45%	kudingcha dicaffeoylquinic acids Low does (LD)−3.3 mg/mouse high dose (HD)-10.0 mg/mouse	Oral gavage	Feces, 16S rRNA, V4 region, Illumina MiSeq:Alpha diversity:Shannon: LD-SH, HD-NS Simpson: LD-SL, HD-NS Beta diversity: PCoA, NMD-LD cluster near HFD, HD cluster relatively far from HFD Phyla:*Firmicutes*-LD, HD-NS *Bacteroidetes*: LD, HD-NS *Proteobacteria*: LD, HD-NS *Actinobacteria*: LD-SL, HD-NS *Verrucomicrobia*: LD-NS, HD-SH F:B ratio-NS Genera:*Akkermansia, Bifidobacterium*, *Anaerobacterium*-LD, HD-SH *Coprobacter, Olsenella*-LD, HD-L		NS	Perirenal: LD, HD-SL Epid:HD-SL				CRP:LD, HD-SL TNFa:LD, HD-SL Il-6:LD, HD-SL	TC:LD-NS, HD-SL TAG:LD, HD-NS LDL: LD, HD-SL HDL: LD-NS, HD-SL	LPS:LD, HD-SL
12	Sung et al., 2017 [47]	C57BL/6N mice, M, 8 w	10/grp NDND + RHFHSHFHs + R	*p*	8 w	45%	Resveratrol (R)-0.4%	Within diet	Cecal content, 16s rRNA, V3 region, Illumina MiSeq: PCA-distinct cluster F:B ratio: SL Genera:*Moryella*-SL *Akkermansia*-SL *Bacteroides*-SH *Parabacteroides*-SH LDA > 3:* Anaerostipes, Adlercreutzia, Parabacteroides, Coprobacillus*		NS	Total body fat: SL	OGTT (AUC): SL					
13	Zhuoqun Liu et al., 2019 [48]	C57BL/6J mice, M, 3 w	7/grp ND HFD HFD + HTHFD + Fecal transplantation (FT) * only results for HT reported in this review	*p*	8 w	45%	Hydroxytyrosol (HT)−50 mg/kg/day	Oral gavage	Feces, 16s rRNA, V3–V4, Ilumina MiSeq: Alpha diversity: Simpson Index-NSBeta-diversity: PCoA-the HT clusters are not distinct from HFD Phyla:*Firmicutes*-NS *Bacteroidetes*-NS *Proteobacteria*-NS *Deferribacteres*-NS F:B-NS Genera:*Lactobacillus*-SH *Rikenella*-SL *Desulfovibrio*-NS *Ruminiclostridium*-NS Species: *Lactobacillus johnsonii*-SH*Anaerotruncus* sp. *G3 [2012]*-SL	NS	NS	RP-SL Epid-SL SAT: NS	FG-SL OGTT(AUC)-NS ITT(AUC)-NS	FI-SL	SL			LPS-SL
14	Ushiroda et al., 2019 [49]	C57BL/6N mice, M, 5 w	8/grp NDHFDHFD + EGCG	*p*	8 w	32%	Epigallocatechin gallate (EGCG)-0.32% within diet	Within diet	Cecal content, 16 s rRNA, V3–V4 region, Illumina MiSeq: Alpha-diversity: Chao 1 index-NSShannon index-SLBeta-diversity: PCoA-distinct cluster Phyla: *Firmicutes*-SL *Bacteroidetes*-NS *Actinobacteria*-SH *Deferribacteres*-SL *Proteobacteria*-SL *Verrucomicrobia*-SH F:B ratio-SL Genera: *Adlercreutzia, Akkermansia, Allobaculum, Parabacteroides, f_Erysipelotrichaceae; g_Clostridium*-SH *Mucispirillum, [Ruminococcus], f_Lachnospiraceae; g_Unclassified, f_Desulfovibrionaceae; g_Unclassified, and Anaerotruncus*-SL	NS	NS	Epid-NS					Serum:TC, TAG, HDL, LDL, NEFA-NS Liver:TAG-SL	
15	Sheng et al., 2018 [50]	C57BL/6 wild-type (WT) mice, M, 3 w	ND WDWD + EGCG WD + vancomycin + polymyxin B + AbxWD + Akkermansia muciniphila supplementation * only results for EGCG reported in this review	T	8 w	21%	Epigallocatechin gallate (EGCG) −100 ug/d/gram body weight	Oral gavage	Cecal content, 16 s rRNA, V4 region, Illumina MiSeq: Phyla: *Firmicutes*-NS *Bacteroidetes*-NS *Proteobacteria*-SL *Verrucomicrobia*-SH *Deferribacteres*-SL *Actinobacteria*-NS Family: *Enterococcaceae*-SH *Verrucomicrobiaceae*-SH *Lachnospiraceae*-SL *Desulfovibrionaceae*-SL *Bacteroidaceae*-SL *Prevotellaceae*-SL *Rikenellaceae*-SL *Deferribacteraceae*-SL	SH	SL	SL	FG-NSITT (AUC)-SL	PYY-SHGLP-1-SH			TC-SL TAG-SL	LPS-SL
16	W. Liu et al., 2017 [51]	C57BL/6 mice, M, 8 w	10–12/grp ND HFDHFD + GSPE HFD + antibiotics + GSPEHFD + antibiotics * only results for GSPE reported in this review	*p*	7 w	60%	300 mg/kg body weight grape seed proanthocyanidin extract (GSPE)	Oral gavage	Feces, 16s rRNA, V3–V4 region, Illumina MiSeq:Alpha diversity(chao1)-NSBeta-diversity (PCoA)-distinct cluster Phyla: *Firmicutes*-NS *Proteobacteria*-SH *Actinobacteria*-H Genera: *Prevotella*-H *Clostridium XIVa*-SH *Escherichia/Shigella*-SH *Blautia*-SH *Flavonifractor*-SH *Arthrobacter*-SH *Roseburia spp*-SH *Roseburia inulinivorans*-SH *Lactococcus*-SL*Bacteroides*-SL		NS	Epid-SLInguinal-NS	ipGTT(AUC)-NSITT(AUC)-SL			TNFa, IL-6, MCP-1-SL		
17	Wang et al., 2019 [52]	ICR mice, M, 5–6 w (29–31g)	6/grp NDHFDHFD + CA	*p*	6 w	18.40%	Chlorogenic acid(CA)-150 mg/kg/day	Oral gavage	Cecal content, 16s rRNA, V3–V4, Ilumina MiSeq:Alpha diversity-NS Phyla: *Firmicutes*-NS*Bacteroidetes*-NS *Proteobacteria*-NS *Verrucomicrobia*-NS *Actinobacteria*-NS F:B-NS Family:*Desulfovibrionaceae, Ruminococcaceae, Lachnospiraceae, Erysipeiotrichaceae*-SL *Bacteroidaceae, Lactobacillaceae*-SH Genera:*Oscillospira, Coprococcus, Anaerotruncus, Allobacterium, Bifidobacterium, Turicibacte*-L*Bacteroides and Ruminococcus*-H		SL	Epid-SL					TC-SL TAG-SL LDL-SL HDL-SH	
18	W. Liao et al., 2018 [53]	C57BL/6J mice, M, 8 w	7–8/grp NDND+RHFD HFD + R HFD + FT * only results for HFD+R included in this review	*p*	4 w	60%	Resveratrol (R) − 400 mg/kg in diet	Within diet	Feces, 16s rRNA, V4–V5 regions, Illumina HiSeq:Alpha-diversity (Shannon)-SL Beta-diversity(PCoA)-distinct cluster Phyla:*Firmicutes*-L *Bacteroidetes*-H *Proteobacteria*-HLDA > 3.5: *Bacteroidaceae, lachnospiraceae, Bacteroides*	NS	SL	Perigonadal-SL Inguinal-SL	OGTT(AUC): SL IpITT (AUC)-SL					
	**PURE COMPOUNDS-RATS**																	
19	L. Zhao et al., 2017 [54]	Wistar rats, M, (160–180 g)	8/grp NDHFD HFD + QR	T	8 w	45%	Combination of Quercetin(Q) − 30 mg/kg body weight/day Resveratrol (R)-15 mg/kg body weight/day	Oral gavage	Feces, 16s rRNA, V4–V5 regions, Illumina MiSeq: Alpha-diversity (Shannon index)-SH Beta-diversity (PCoA)-distinct cluster Phyla:*Firmicutes*–SL *Bacteroidetes*-NS *Proteobacteria*–SH *Verrucomicrobia*-NS *Actinobacteria*-NS F:B ratio-NS Genera: *Ruminococcaceae_UCG-014*-SH *Bacteroidales_S247_group_norank*-SH*Ruminococcaceae_UCG-005*-SH *[Eubacterium]_coprostanoligenes_group*-SH *Akkermansia*-SH *Lachnoclostridium*–SL *Bilophila*-SL	NS	SL	Perinephric-SLEpid-SLSAT: NS		SL		Leptin-NSAdiponectin-SHTNFa-SL IL-6-SL MCP-1-SL	TC-SL TAG-SL HDL-SH LDL-SL	
20	C. Yang et al., 2019 [55]	Wistar rats, M, 5 w	6/grp NDHFDHFD + RHFD + SHFD + C	*p*	8 w	45%	Resveratrol(R) − 400 mg/kg of diet Sinapic acid (S) − 200 mg/kg of dietR-S(C) − 400 mg/kg resveratrol and 200 mg/kg sinapic acid	Within diet	Feces, 16s rRNA, V3–V4 regions, Illumina MiSeq: Alpha-diversity (ACE, Chao1): R, S, C-SHNon-metric multi-dimensional scaling (NMDS)-distinct cluster for R, S Phyla: *Firmicutes*; R, S-NS, C-SH *Bacteroidetes*: R, S, C-NS *Proteobacteria*: R, S, C-NS *Tenericutes*: S, C-SL, R-NS *Actinobacteria*: R-NS, S, C-SL Genera: Unclassified Peptostreptococcaceae: R, S, C-NSRF-39: S, C-SH Blautia: R, S-SHDorea: R, S-SH Ruminococcaceae:R-SH, C-SLRoseburia: C-SH Clostridiales, Ruminococcus, Oscillospira, Lachnospiraceae, S24_7, Bacteroides, Desulfovibrionaceae: R, S, C-NS	R, S, C-NS	R, S, C-NS		FG: R-SL OGTT(AUC): R, S, C-NS	FI-R, S, C-NS	R, C-SL		TC: R, S, C-NS TAG:S-SL HDL:R-SH LDL:R, S, C-NS NEFA:R, S, C-NS	
21	Luo et al., 2019 [56]	Sprague Dawley rats, M, 5 w	16/grp ND HFDHFD + LSIFHFD + HSIF	T	4 w	ND + 15%pork fat	LSIF = 150 mg/kg soy isoflavone (SIF)HSIF = 450 mg/kg(SIF)	Oral gavage	Cecal content, 16S rRNA, V3–V4 region, Illumina HiSeq:Beta-diversity:PCA-no distinct pattern in treatment grps Phyla: *Firmicutes*:LSIF, HSIF-SL *Bacteroidetes*:LSIF, HSIF-SH *Fusobacteria*:LSIF, HSIF-NS *Actinobacteria*:LSIF, HSIF-NS *Proteobacteria*:LSIF, HSIF-SH F:B ratio:LSIF, HSIF-SL Genera: *Coprococcus_1:LSIF, HSIF*-SH *Faecalibacterium:LSIF, HSIF*-SH *[Eubacterium]_oxidoreducens group:HSIF*-SH*Ruminococcaceae UCG-005:HSIF*-SH *Phascolarctobacterium:LSIF, HSIF*-SH *Prevotella_9:LSIF, HSIF*-SH *Lachnospira:LSIF, HSIF*-SH *Bacteroides:LSIF, HSIF*-SH *Ruminiclostridium_9:HSIF)-SH, LSIF*-NS *[Eubacterium]_ruminantium group: LSIF-SH, HSIF*-SL *Morganella:LSIF, HSIF-SL* *Lactobacillus:LSIF, HSIF*-SL *Oscillibacter:HSIF-SL Ruminococcaceae_NK4A214:LSIF, HSIF*-SL *Dorea:LSIF, HSIF*-SL *Pasteurella:LSIF, HSIF*-SL *Blautia:LSIF, HSIF*-SL *Roseburia:LSIF, HSIF*-NS *Candidatus Saccharimonas:LSIF, HSIF*-NS *Ruminococcus_1:LSIF, HSIF*-NS	LSIF, HSIF-NS	LSIF, HSIF-SL						TC:LSIF-NS, HSIF- SL TAG:LSIF, HSIF-NS HDL:LSIF-NS, HSIF-SL LDL:LSIF-NS, HSIF-SL	LPS: LSIF, HSIF-SL
	**COMBINATION OF PURE COMPOUNDS**																	
22	Yong-Feng et al., 2019 [57]	C57BL/6J mice, M, 8 w	12/grp NDHFDHFD + TFQ	*p*	12 w	60%	Total flavonoids of Quzhou (TFQ)-300 mg/kg/day	Oral gavage	Colonic content, 16S rRNA, V3–V4 regions, Illumina HiSeq: Alpha diveristy-Chao1 index and the Shannon index-SH Beta-diversity (PCoA)-TFQ group was closer to HFD Phyla:*Firmicutes*-NS*Verrucomicrobia*-SH *Bacteroidetes*–SH *Actinobacteria*-SL F:B ratio-SL Genera:*Akkermansia*-SH *Alistipes*-SH *Dubosiella*-SL *Faecallbaculum*-SL *Lactobacillus*-SL LDA > 4 *Blautia*		SL	Epid-SL	FG-SL OGTT(AUC)-SL ITT(AUC)-NS	FI-NS	SL		Serum: TC-SL TAG-SL LDL-SL HDL-NS NEFA-SL Liver:TC-NS TAG-SL	LPS-SL
23	Zhu et al., 2018 [58]	Sprague-Dawley rats, M (120–140 g)	6/grp NDND+LPT ND + MPT ND + HPTHCHC + LPTHC + MPT HC + HPT	T	2wks	HC diet-81.8% basic diet, 6% dried egg yolk, 5% full cream milk powder, 5% lard, 2% cholesterol, and 0.2% sodium cholate	Low persimmon tannin (LPT)-50 mg/kg bwMedium persimmon tannin (MPT)-100 mg/kg bwHigh persimmon tannin (HPT)-200 mg/kg bw	Oral gavage	Cecal content, 16s rRNA, v4 region, Illumina MiSeq: Phyla:*Firmicutes*, *Bacteroidetes*, *Proteobacteria*-NS F:B ratio-MPT, HPT-SL Genera:*Roseburia*: LPT-SL *Helicobacter*: LPT-SL *Bacteroides*: LPT-SL *Oscillospira*: LPT-SH *Phascolarctobacterium*: LPT, MPT, HPT-NS *Ruminococcus*: LPT, MPT, HPT-NS *Sutterella*: LPT, MPT, HPT-NS *Desulfovibrio*: LPT, MPT, HPT-NS *Prevotella*: LPT, MPT, HPT-NS	LPT, MPT, HPT-NS	LPT, MPT, HPT-NS						Serum:TC: LPT, HPT-SL TAG:MPT- SL LDL: LPT-SL Liver:TC: LPT, HPT, MPT-NS TAG:LP, HPT, MPT-NS	
	**PHENOLIC EXTRACTS-MICE**																	
24	H. Lee et al., 2019 [59]	C57BL/6Nmice, M, 5 w	*n* = NANDHFDHFD + RO125HFD + RO250	T	16 w	45%	Rubus occidentalis(RO) 125: 125 mg/kg/day; RO250: 250 mg/kg/day	Oral gavage	Cecal content, 16s rRNA, V4 region, Illumina MiSeq, Alpha diversity (Chao1, Shannon)-NS Beta diversity PCoA-clear separation between groups Phyla: *Bacteroidetes*:RO125, RO250-H *Deferribacteres*:RO125, RO250-H F:B ratio:RO125, RO250-L Genera:*Butyricimonas*: RO250-SH *Bacteroides*:RO250-SH *Mucispirillum*:RO250-SH *Ruminococcus*:RO250-SH *Oscillospira*:RO125-SH Species: *Mucispirillum schaedleri*:RO250-SH		NS		iPGTT (AUC)RO250-SL					
25	Collins et al. 2016 [60]	C57BL/6J mice, M, 4 w	10/grp ND HFDHFD + EPHFD + NEPHFD + EP-NEPHFD + GP	*p*	16 w	44%	Extractable polyphenol (EP)-1.1 g/kg of dietNon-extractable polyphenol (NEP)-3.5 g/kg of dietEp-NEP-1.1 g.kg EP 3.5 g/kg NEP of dietGrape powder (GP)-50 g/kg of diet	Within diet	Cecum mucosa, 16s rRNA, V4–V5 regions, Illumina MiSeq: Observed species (AUC):EP-NSNEP-NS EP-NEP-NSGP-SHBeta-diversity (PCoA)-No distinct cluster Genera: *Coprococcus*: NEP, EP + NEP-SH *Ruminococcus*: EP, NEP, EP-NEP, GP-NS *Rc4-4*: NEP, EP-NEP, GP-SL *S24-7*: EP-NEP-SL *Adlercreutzia*; EP, NEP, EP-NEP, GP-NS*HRF-39*: NEP-SH		EP-SLNEP-SL EP-NEP-SL GP-NS	% body fat (wk 15): EP-SL NEP-NS EP-NEP-SL GP-NS WAT:EP–SL NEP-SL EP-NEP-SL GP-NS	IpGTT (AUC):EP-SL NEP-SL EP-NEP-SLGP-NS		EP-SLNEP-NS EP-NEP-SLGP-NS	Plasma MCP-1: NEP-NS EP-NEP-SLGP-NS	Plasma TAG:EP-SL NEP –NS EP-NEP-SL GP-NS Liver TAG:EP-Ns NEP-NS EP-NEP-SL GP-NS	Plasma LBP:EP –NS NEP-SL EP-NEP-SL GP-NS
26	Guo et al., 2019 [61]	C57BL/6 mice, M, 3 w	Study 1, *n* = 9-12/grp NDND + BEHFDHFD + BE The results of study 2 and 3 are not included in this review	*p*	14 w	60%	ND+BE-5 g/L blueberry extract (BE) in drinking waterHFD + BE-0.5% (*m/v*) BE in drinking water	Drinking water	Feces, 16s rRNA, Illumina HiSeq: Alpha-diversity:Shannon index-SH Beta diversity (PCoA)-pattern similar to ND Phyla:F:B ratio-SL Genera:*Akkermansia*-SH *Bifidobacterium*-SH *Desulfovibrio*-SL *Bilophila*-SL	NS	SL	Epid-SL Inguinal-SL	OGTT(AUC)-SL ITT(AUC)-SL			TNF-a-SLIL-6-SL Leptin-SL	Plasma:TAG-SL Liver:TAG-SL	LPS-SL
27	D. M. Cheng et al., 2016 [62]	C57BL/6J mice, M, 5 w	15/grp ND HFDHFD + RSL HFD + GL	*p*	13 w	60%	Rutgers Scarlet Lettuce (RSL), Green lettuce (GL)-6.4% (*w/w*) RSL or GL powder	Within diet	Feces, cecal content, 16s rRNA, V3–V4 regions, Illumina Miseq:PCA-same diet clustered together, HFDs, ND and treatments groups formed distinct cluster Phyla: *Firmicutes*: RSL-L *Bacteroidetes*: RSL-H *Verrucomicrobia*: RSL, GL-NS *Proteobacteria*: RSL, GL-NS *Actinobacteria*: RSL, GL-NS *Tenericute*: RSL, GL-NS F:B ratio: RSL, GL-NS Genera (fecal and cecal content):*Roseburia spp*: RSL-SH *Ruminococcus spp*:RSL-SH *rc4_4*:RSL-SH *Coprococcus*:GL-SH *Blautia*:GL-SH *Moryella spp*:GL-SL *Clostridium spp*:GL-SH		RSL, GL-NS	Fat mass:RSL, GL-NS	FG: RSL, GL-NS OGTT(AUC)-RSL-SL, GL-NS(wk9) ITT(AUC)-RSL, GL-NS	FI: RSL, GL-NS			TAG:RSL, GL-NS FFA:RSL, GL-NS	LPS:RSL, GL-NS
28	Zhibin Liu et al., 2016 [63]	C57BL/6J mice, M, 10 w	10/grp ND HFD HFD + GTHFD + OT HFD + BT	*p*	13 w	45%	Green tea (GT), oolong tea (OT), black tea (BT)-dosage:NA	Drinking water	Cecal content, 16s rRNA, V3–V4 regions, Illumina MiSeq:Alpha diversity: ACE, Chao1: GT, OT, BT-SH Shannon: GT, BT-SH Beta-diversity (PCA)-distinct clusters between ND, HFD, HFD + tea (all tea) Taxa (LDA > 3.5): Family/Genera:*S24-7, Blautia sp., Helicobacter ganmani, Oscillibacter sp*., *Anaerotruncus sp*.-SH *Alistipes sp., Lachnospiraceae (OTU173), Lachnospiraceae (OTU45), S24-7, Akkermansia sp., Rikenella microfusus*-H*llobaculum sp., Bacteroides acidifaciens, S24-7(OTU319), S24-7(OTU192), Lachnospiraceae, S24-7 (OTU535), Clostridium leptum (OTU450), Parabacteroides goldsteinii*-L		GT, OT, BT-SL	VAT: GT, OT, BT-SL	FG: GT, OT, BT-SL				TC: GT, OT, BT-SL TAG: GT, OT, BT-SL LDL: GT, OT, BT-SL HDL: GT, OT, BT-SL	LBP: GT, OT, BT-SL
29	Griffin et al., 2017 [64]	C57BL/6J mice, M, 9 w	8/grp NDHFDHFD10HFD100	T	12 w	45%	HFD10 ∼10 grape seed extract (GSE) mg/kg/day HFD100 ∼100 mg/kg/day	Within diet	Mucosal adherent microbiota-small intestine, cecum, colon), 16S rRNA, V4, Illumina MiSeq (only for HFD10): Phyla:*Firmicutes*–NS *Bacteroidetes*-NS *Proteobacteria* –NS *Deferribacteres*-NS F:B ratio-NS Genera:Cecum:*Allobaculum*-SL *Lactococcus*–SL Colon:*Turicibacter spp.*-SH *Phascolarctobacterium*-SH *Roseburia*-SH *Peptoniphilus*-SH *Desulfovibrionaceae spp*-SH	HFD10-NS HFD100-NS	HFD10-NS HFD100-NS	Total body fat:HFD10-NS HFD100-NS	FG:HFD10-SL HFD100-NS OGTT(AUC): HFD10-SL HFD100-NS ITT (AUC):HFD10-NS HFD100-NS					
30	Y. Li et al., 2019 [65]	C57BL/6J (B6) mice, M, (22 ± 2 g)	10/grp NDHFDHFD + CROHFD + SRO	*p*	12w	24.50%	Common rapeseed oil (CRO)-10%in the dietRapeseed oil with sinapine (SRO)-CRO with 100 mg sinapine in the diet	Within diet	Feces, 16S rRNA, V3–V4 region, Illumina MiSeq:Alpha diversity:Chao1 and Shannon index: CRO-NS, SRO-SL Simpson diversity: CRO-NS, SRO-SL Beta-diversity: PCoA-distinct clustering of each SRO, CRO overlap with HFD Phyla:*Firmicutes, Bacteroidetes*: CRO-NS, SRO-L *Proteobacteria*: CRO-NS, SRO-L Genera:*Muribaculaceae, Desulfovibrio, Lachnospiraceae*-CRO-H *Mucispirillum*: CRO-L *Lactobacillus*, *Bifidobacterium*: SRO-H *Blautia*:SRO-SH *Mucispirillum*: SRO-L(LDA) effect size (LEfSe) > 3: CRO-*Ruminiclostridium*SRO-*Blautia*, *Desulfovibrio*	NS	CRO-NS SRO-SL	Epid: CRO-NS SRO-SL Perirenal:CRO-NS SRO-SL	FG:CRO-NS SRO-SL	FI:CRO-NS SRO-SL	CRO-NS SRO-SL		Serum: TAG:CRO-NS, SRO-SL LDL: CRO, SRO-SL Liver: VLDL: CRO-NS, SRO-SL	
31	Ma et al., 2019 [66]	C57BL/6 mice, M, 6 w	8/grp ND HFDHFD + TPLHFD + TPMHFD + TPH	*p*	12 w	36.71%	Tea polyphenol lowdose (TPL)-100 mg/kg/day Tea polyphenol medium dose (TPM)-200 mg/kg/day Tea polyphenol high dose (TPH)-400 mg/kg/day	Oral gavage	Cecal content, 16S rRNA, V3–V4 region, Ion S5 XL platform: Phyla:*Firmicutes*:TPL, TPM, TPH-NS *Bacteroidetes*:TPL, TPM, TPH-L *Proteobacteria*:TPL, TPM, TPH-SL *Actinobacteria*:TPL, TPM, TPH-H *Verrucomicrobia*:TPL, TPM, TPH-H Genera:TPL: *Butyrivibrio, Anaerostipes*, *Alloprevotella*-SHTPM: *Paraprevotella*-SHTPH: *Alitipes, Bacteroides, Faecalibaculum, Erysipelatoclostridium, Flavonifractor, Coprobacillus, Fusicatenibacter, Parasutterella, Bifidobacterium, Akkermansia, Ruminococcaceae, Lachnoclostridium, Clostridiales, Roseburia*, *Blautia*-SHLDA > 4: TPL: *unidentified_Lachnospiraceae, Alloprevetella, Anaerostipe* TPM: *Family Atopobiaceae* TPH:*Lachnoclostridium, Akkermansia, Bifidobacterium, Erysipelatoclostridium, and unidentified Clostridiales*		TPL, TPM, TPH-SL		FG:TPL, TPM, TPH-NS	FI:TPL, TPM-SL, TPH-NS	TPL.TPM-SL, TPH-NS	TNF-α: TPL, TPM, TPH-SLIL-6: TPL, TPM, TPH-SL	TC:TPL, TPM, TPH-NS TAG:TPL, TPM, TPH-NS HDL:TPL, TPM, TPH-SH LDL:TPL-NS, TPM, TPH- SL	LPS:TPL, TPM-NS, TPH-SL
32	Van Hul et al., 2018 [67]	C57BL/6J mice, M, 9 w	14/grp NDHFDHFD + CBE HFD + GPE	*p*	8 w	60%	Cinnamon bark extract (CBE)-2 g/kg grape pomace extract (GPE)-8.2 g/kg	Within diet	Feces, 16s rRNA, V3–V4 regions, Illumina MiSeq: Beta-diversity (PCoA): Most CBE and GPE-fed mice were separated from the untreated HFD mice according to the axis 2. Phyla (CBE and GP): *Firmicutes, Bacteroidetes, Actinobacteria, Proteobacteria*-NS Genera: CBE:*Peptococcus*-SL GPE: *Desulfovibrio*-SL *Clostridium sensu stricto*-SL *Lactococcus*-SL *Allobaculum*-SH *Roseburia*-SH	CBE, GPE-NS	CBE, GPE-NS	VAT: CBE, GPE-NS SAT: CBE, GPE-NS	OGTT (AUC): CBE-SL GPE-NS	FI:CBE, GPE-NS	IR-index: CBE-NS GPE-SL	Leptin:CBE, GPE-NSResistin: CBE, GPE-NS IL-1B:CBE- SH GPE-NS IGNy: CBE, GPE-NS MCP1:CBE- SH GPE-NS MIP1A:CBE, GPE-NS PAI1: CBE, GPE-NS	Plasma:TC, TAG, NEFA:CBE, GPE-NS Liver:TC:CBE, GPE-NS TAG-CBE-NS, GPE-SL	
33	Anhê et al., 2015 [68]	C57Bl/6J mice, M, 8 w	12/grp NDHFHS HFHS + CE	*p*	8 w	65%	Cranberry powdered extract (CE)-200 mg/kg	Oral gavage	Feces, 16s rRNA, V6–V8 regions, 454 pyrosequencing:Beta diversity-PCA-distinct cluster at week 5 and 9 Phyla: *Firmicutes*–NS *Bacteroidetes*-SL at wk 9 compared to wk1(within CE)*Verrucomicrobia*-SH at wk 9 compared to wk 1 (within CE) Genera (wk 9 vs. wk 1-within CE): *Akkermansia*–SH *Oscillibacter*–SH *Ruminococcus*-SH *Pseudoflavonifractor*-SH unclassified *Ruminococcaceae*-SH unclassified *Porphyromonadaceae*-SH *Barnesiella*-SL unclassified *lachnospiraceae*-SL *Turicibacter*-SL*Eubacterium*-SL *Clostridium*-SL*Lactobacillus*-SL	SL	SL	VAT: SL SAT: NS	FG-NS OGTT(AUC)-NS ITT(AUC):SL	FI-SL C-peptide AUC-SL	SL		Plasma:TC-SL TAG-SL Liver:TAG-SL Jejunum:TAG-SL	SL
34	Anhê et al., 2017 [69]	C57Bl/6J mice, M, 8 w	8–11/grp ND ND + CEHFHSHFHS + CE	T	8 w	65%	Cranberry extract (CE)-200 mg/kg	Oral gavage	Feces, 16s rRNA, V3–V4, Illumina MiSeq:Beta-diversity (PCoA)-distinct cluster for CE F:B ratio-SL Genera LDA > 2.5: *A. muciniphila, Coprobacillus*, and *Barnesiella*-H	NS	NS	VAT: NS SAT: NS	OGTT (AUC): NS ipITT (AUC):SL				Plasma: TAG-NS Liver:TAG-SL	
35	Anhê et al., 2018 [70]	C57BL/6 J mice, M, 8 w	12/grp NDHFHSHFHS + BBEHFHS + CLEHFHS + CRE HFHS + ABEHFHS + LGE	*p*	8 w	65%	Bog blueberries (BBE), cloudberries (CLE), crowberries (CRE), alpine bearberries (ABE) and lingonberries (LGE)-200 mg powdered extract/kg body weight	Oral gavage	Feces, 16s rRNA, V3–V4 regions, Illumina MiSeq:Beta-diversity (PCA)-distinct cluster for CLE and ABE Phyla: *Firmicutes*-All-NS *Bacteroidetes*-All-NS *Actinobacteria*-All-NS *Proteobacteria*: CLE-SH, ABE, LGE-NS *Tenericutes*-All-NS *Verrucomicrobia*-All-NS F:B ratio-NS Genera (LDA > 2):CLE:*Turicibacter, Akkermansia* (SH), *Bifidobacterium Lactobacillus* (SL) ABE: *Oscillibacter, A. muciniphila* (SH) LGE: *Oscillibacter, Turicibacter* (SH)	All grps-NS	All grps-NS	VAT: All grps-NS SAT: All grps-NS	FG-NS (all) OGTT (AUC)-NS (all) ITT(AUC): CLE-SL C	FI:CLE, ABE, LGE-SLpeptide: All-NSAll (AUC)-NS			Plasma:TAG: CLE, ABE, LGE-SL Liver: TAG: CLE, ABE, LGE-SL	LPS:CLE, ABE, LGE-SL
36	Chen et al., 2018 [71]	C57BL/6 mice, M, 6 w	8/grp ND HFDHFD + KDCHFD + FBT	*p*	8 w	45%	Kudingcha (KDC) and Fuzhuan Brick Tea (FBT)-400 mg/kg/d	Intragastric gavage	Feces, 16s rRNA, V4 region, Illumina MiSeq:Alpha-diversity: Shannon: KDC, FBT-SHSimpson: KDC, FBT-SLInvSimpson: KDS-SH, FBT-NS Beta-diversity-PCoA-distinct clusters of treatment groups Phyla:*Firmicutes*: KDC-NS, FBT-SL *Bacteroidetes*: KDC-NS, FBT-SHF:B ratio: KDC-NS, FBT-SL Genera:*Pseudoflavonifractor*: FBT-SL *Coprobacter*:FBT-SL *Olsenella*: KDC, FBT-SL *Oscillibacter*:FBT-SL *Anaerobacterium*: KDC-SH, FBT-SL *Anaerotruncus*:FBT-SL *Bilophila*:FBT-SL *Clostridium_IV*:FBT-SL, *Streptococcus*: KDC, FBT-SL *Lactobacillus*:FBT-SL, *Lactonifacto*:-FBT-SL *Streptococcus*: KDC, FBT-SL *Leuconostoc*: KDC, FBT-SL *Clostridium_XlVb*:FBT-SL *Anaerotruncus*:FBT-SL *Catabacter: KDC,* FBT-SH *Barnesiella*: KDC, FBT-SH *Alistipes*: KDC, FBT-SH, *Odoribacter*-KDC, FBT-SH *Bacteroides*: KDC, FBT-SH	NS	KDC, FBT-SL	Epid: KDC, FBT-SL Perirenal: KDC, FBT-SL				TNFa:KDC, FBT-SL IL6:KDC, FBT-SL CRP: KDC, FBT-SL	Serum:TC: KDC-NS FBT-SL TAG: KDC, FBT-NS LDL: KDC-NS, FBT-SL HDL: KDC, FBT-NS Liver:TAG: KDC-NS, FBT-SL	LPS:KDC, FBT-SL
37	J. Xu et al., 2019 [72]	C57BL/6 mice, M, 7 w	12/grp ND HFDHFD + J	*p*	8 w	60%	Jamun extract (J)-100 mg/kg	Oral gavage	Feces, 16s rRNA, V3–V4regions, Illumina HiSeq: Alpha-diversity (Shannon, chao1)-NSBeta-diversity (PCoA)-distinct cluster Phyla: *Firmicutes*-L*Bacteroidetes*-H F:B ratio-SL Genera:*Bacteroides, Alistipes, Prevotella, Alloprevotella*-H *ClostridiumXlVb*-L	NS	SL	VAT:SL SAT:SL	FG-SL OGTT (AUC): SL ITT (AUC)-SL	FI-SL	SL		Plasma:TC-NS TAG-SL FFA-SH Liver: TC-SL TAG-SL FFA-SL	
38	Dey et al., 2019 [73]	C57BL/6J mice, M, 5 w	10/grp ND ND + GTEHFDHFD + GTE	*p*	8 w	60%	Green tea extract (GTE)-2% (*w/w*)	Within diet	Cecal content, 16s rRNA, V4–V5, Ilumina MiSeq:Alpha-diversity: Shannon index and the Chao1-SH Beta-diversity:PCA-cluster closer to ND Phyla (significance not mentioned):*Firmicutes*-L*Bacteroidetes*-H *Actinobacteria*-H *Verrucomicrobia*-H *Proteobacteria*-unaffected *Tenericutes-unaffected* F:B ratio-NS Genera:*Bifidobacterium*-H*Blautia*-H *Dorea*-H *Lactobacillus*-H *Ruminococcus*-H *SMB53*-L *Akkermansia*-SH Species:*Akkermansia muciniphila*-SH *Ruminococcus gnavus*–H *Bifidobacterium pseudolongum*-SH *Bifidobacterium adolescentis*-SH	SH	SL	Epid-SL RP-SL SAT: SL	FG-NS	FI-NS	SL		TC-SL TG-SL NEFA-SL	LPS-NS FITC-dextran-SL
39	S. Wu et al., 2018 [74], M. Liu et al., 2018 [75]	C57BL/6N mice, M, 5 w	4/grp NDND + 1% LCBPHFDHFD + 0.5% LCBPHFD + 1% LCBP	*p*	45 d	40%	Lonicera caerulea L. Berry Polyphenols (LCBP)-0.5% and 1%	Within diet	Feces, 16S rRNA, V3–V4 regions, Illumina MiSeq:Alpha-diversity: Chao1, Shannon index, PD-NS Phyla (both doses):*Firmicutes*-L*Bacteroidetes*-H *Proteobacteria*-H F:B ratio-L Genera:Both doses:Bacteroides-H Parabacteroides–H *Staphylococcus, Lactobacillus, Oscillospira. Ruminococcus*-L		0.5%, 1% LCBP-SL		FG: 0.5%, 1% LCBP-SL	FI 0.5%, 1% LCBP-SL	0.5%, 1% LCBP-SL	IL-2: 0.5%, 1% LCBP–SL IL-6: 0.5%, 1% LCBP-SL MCP1: 0.5% LCBP –NS 1% LCBP-SL TNFa: 0.5%, 1% LCBP-SL	TAGSerum: 0.5% LCBP-SL 1% LCBP-SL Liver: 0.5% LCBP –SL 1% LCBP-SL	Endotoxin: Serum: SL (0.5%, 1% LCBP) Liver: SL (0.5%, 1% LCBP)
40	C. Wu et al. 2019 [76]	C57BL/6J mice, M, 8 w	8/grp NDHFDHFD + PTF HFD + AbHFD + Ab + PTF * only results for PTF reported in this review	T	6w	60%	Pandanus tectorius fruit extract (PTF)-200 mg/kg bw	Oral gavage	Feces, 16S rRNA, V4–V5 regions, 454 FLX pyrosequencing platform: Alpha-diversity(Shannon, Choa1)-SHBeta-diversity (PCA)-distinct cluster Phyla:*Firmicutes*-SH *Bacteroidetes*-SL *Actinobacteria*-SH *Verrucomicrobia*-NS *Proteobacteria*-NS *Tenericutes*-NS Genera:*Lactobacillus*-SH *Lactococcus*-SH *Streptococcus*-SH *Enterococcus*-SH *Clostridium sensu stricto*-SH*Bacteroides*-SL *Alistipes*-SL *Akkermansia*-SL *Clostridium XIVa group*-SL	NS	SL	Epid–NS SAT: NS	FG-SL OGTT (AUC): SL				TC-SL TG-SL LDL-SL	
41	Vezza et al., 2019 [77]	C57BL/6 J mice, M, 5w	9/grp NDND + OL HFDHFD + LDHFD + MDHFD + HD FT * only results for LD, MD, HD reported in this review	NA	5 w	60%	ND + olive leaf (OL)-25 mg/kgHFD + LD-1 mg/kgHFD + MD-10 mg/kgHFD + HD-25 mg/kg	Oral gavage	Feces, 16S rRNA, V4–V5, Illumina MiSeq:Beta-diversity: PCA-distinct pattern Phyla:*Firmicutes*: LD, MD-NS, HD-SL *Bacteriodetes*: LD-SL, MD-NS, HD-SH *Proteobacteria*: LD-SH, MD-SL, HD-NS *Actinobacteria*: LD, MD, HD-NS *Verrumicrobioa*: LD-SH, MD-SL, HD-NS *Tenericutes*: LD-NS, MD, HD-SHF:B ratio: LD-NS, MD, HD-SL Genera: *Cytophaga*: LD, MD-NS, HD-SH *Akkermansia*: LD-NS, MD, HD-SH	LD, MD, HD-NS	LD, MD, HD-SL	Epid:LD, MD, HD-SL	FG:LD, MD-NS, HD-SL OGTT(AUC):LD, MD, HD-SL	LD, MD, HD-SL	LD, MD, HD-SL		LDL:LD-NS, MD, HD-SL HDL: LD, MD, HD-NS	
42	Henning et al., 2018 [78]	C57BL/6J mice (strain JAX 000664), M, 6–7 w	12/grp NDHFHSHFHS + GTPHFHS + BTP	*p*	4 w	NA	0.5 g/100 g of diet providing 0.25 g polyphenols/100 g diet of green tea polyphenol (GTP) or black tea polyphenol (BTP)	Within diet	Cecal content, 16s rRNA, v4 region: Beta-diversity (PCoA)-distinct cluster for GTP, BTP Phyla:*Firmicutes*: GTP, BTP-SL *Bacteroidetes*: GTP, BTP-SH *Actinobacter*: GTP, BTP-SL Genera:*Parabacteroides, Bacteroides, Prevotella*: GTP, BTP-SH *Roseburia, Lactobacillus, Blautia, Anaerostipes, Shuttleworthia, Bryantella, Lactococcus, Acetitomaculum, Collinsella*: GTP, BTP-SL *Clostridium Coprococcus*: GTP-SH *Turicibacter, Marvinbryantia*: GTP-SL *Oscillibacter, Anaerotruncus, Pseudobutyrivibrio*: BTP-SH	GTP-SL, BTP-NS	GTP, BTP-SL	Mesen:GTP, BTP-SL Epid: GTP, BTP-SL SAT: GTP, BTP-NS						
	**PHENOLIC EXTRACTS-RATS**																	
43	R. Zhao et al., 2019 [79]	Sprague-Dawley rats, M, (250–270 g)	12/grp NDHFD HFD +P PPLHFD + PPPH	*p*	12 w	45%	PPPL-150 mg/kg of Pomegranate polyphenols (PPP) PPPH-300 mg/kg of PPP	Oral gavage	Feces, 16s rRNA, V4–V5 regions, Illumina HiSeq:Beta diversity (PCoA)-some PPPL and PPPH separated from HFD but some not. Phyla: *Firmicutes, Bacteroidetes, Proteobacteria, Tenericutes, Actinobacteria*-no significance were given F/B ratio:PPPL-SL Genera: HFD vs. PPL:*Bacteroidales S24-7 group_norank*-SH *Paraprevotella*-SH *Lactobacillus*-SH *Family XII AD3011 group*-SL *Lachnospiraceae_uncultured*-SL *Ruminococcaceae_uncultured*-SL *Ruminococcaceae UCG-009-SL Ruminococcus 1*-SHHFD vs. PPPH: *Lactobacillus*-SH *Family XII AD3011 group*-SL *Lachnospiraceae_uncultured*-SL *Prevotellaceae UCG-001*-SH	PPPL, PPP-NS	PPPL, PPP-SL					TNFa:PPPL, PPPH-SL IL-6: PPPL-NS, PPPH-SL IL-1B:PPPL-SL, PPPH-NS	TC:PPPL-SL, PPPH-NS TAG:PPPL-NS, PPPH-SL HDL:PPPL, PPPH-NS LDL:PPPL, PPPH-SL	LPS:PPPL-SL, PPPH-NS
44	S. Lee et al., 2018 [80]	Wistar rats, M, (200-220g)	8/grp NDHFDHFD + BB	*p*	8 w	45%	HFD with 10 g freeze-dried blueberry powder (BB)/100 g	Within diet	Cecal content, 16s rRNA, V4 region, Illumina MiSeq:Phyla:* Firmicutes*-SL*Bacteriodetes*-SL *Proteobacteria*–SH *Fusobacteria*–SH Genera:*Actinobacillus*-SH *Aggregatibacter*-SH		NS	VAT: NS	OGTT(AUC):NS					Serum LBP-SL
45	H. Xu et al., 2019 [81]	Sprague-Dawley rats, M, 5 w	6/grp NDHFDHFD + PFELHFD + PFEHHFD + CAE(Positive control) * only results for PFEL, PFEH reported in this review	*p*	8 w	ND-76% + fat-12%	PFEL-0.4% Pyracantha fortuneana extract (PFE) PFEH-1% PFECAE-0.4% Citrus aurantium extract (CAE)	Within diet	Feces, 16s rRNA, V4–V5 region, Illumina MiSeq:PFEL and PFEH-combinedAlpha-diversity: Chao1 index-Shannon, and Simpson-NS Phyla:*Firmicutes *–SL *Bacteroidetes*-NS *Actinobacteria*-SH F:B ratio-SL Genera:*Bacteroides, Corynebacterium, Lactobacillus, Blautia*-H*R* *uminococcus, Oscillospira, Flexispira*-SL	PFEL, PFEH-NS	PFEL, PFEH-SL	Epid/bw:PFEL-NS PFEH-SL	FG:PFEL, PFEH-SL				TC:PFEL-NS, PFEH-SL TAG:PFEL-NS, PFEH-SL LDL:PFEL-NS, PFEH-SL HDL:PFEL-NS, PFEH-SH	

M: male; wks: weeks; ND: normal-diet; HFD: high-fat diet; mths: months; *p*: prevention; NA: not available; H: high; L: low; SL: significantly low; SH: significantly high; SAT: subcutaneous adipose tissue; VAT: visceral adipose tissue; FG: fasting glucose; NS: not significant; IpGTT: intraperitoneal glucose tolerance test; AUC: area under curve; HOMA-IR: homeostatic model assessment-insulin resistance; TC: total cholesterol; TAG: triacylglyceride; LDL: low-density lipoprotein; HDL: high-density lipoprotein; LPS: lipopolysaccharide; LDA: linear discriminant analysis; IL-6: interleukin-6; TNFα: tumour necrosis factor alpha; IFNγ: interferon gamma; IL-2: interleukin-2; IL-4: interleukin-4; grp: group; HFHS: high-fat high-sucrose; rRNA: ribosomal ribonucleic acid; PCoA: principal coordinate analysis; mesen: mesenteric; epid: epididymis; FI: fasting insulin; Loxp: locus of X-over P1; OGTT: oral glucose tolerance test; ITT: insulin tolerance test; F:B: *Firmicutes:Bacteroidetes* ratio; NMDS: non-metric multidimensional scaling; GIP: gastric inhibitory polypeptide; GLP-1: Glucagon-like peptide-1; GLP-2: Glucagon-like peptide-2; FITC-dextran: Fluorescein isothiocyanate–dextran; LD: low-dose; MD: medium-dose; HD: high-dose; IL-1: interleukin-1; IL-10: interleukin-10; T: treatment; CRP: c-reactive protein; TBF: total body fat; RP: retroperitoneal; NEFA: non-esterified fatty acid; Abx: antibiotic mix; PYY: peptide tyrosine tyrosine; MCP-1: monocyte chemoattractant protein 1; PCA: principal component analysis; HC: high-cholesterol; *m*/*v*: mass/volume; FFA: free fatty acid; OTU: operational taxonomy unit; IR-index: insulin-resistance index; *w*/*w*: weight/weight; Il-1β: interleukin-1 beta; bw: body weight.

## Supplementary Materials

The following are available online at https://www.mdpi.com/2304-8158/10/2/299/s1, Figure S1. Search strategy (PubMed); Figure S2. Risk of bias using SYRCLE’s Risk of Bias tool; Figure S3. Study quality using Gold Standard Publication Checklist, Figure S4. Effect of polyphenols on energy/food intake, and body weight; Figure S5. Effect of polyphenols on adiposity; Figure S6. Effect of polyphenols on lipid profile; Figure S7. Effect of polyphenols of glucose homeostasis; Figure S8. Adipocytokines, C-Reactive Protein (CRP), and LPS/LBP; Figure S9. Beta-diversity of (a) pure phenolic compounds and (b) phenolic extracts; Table S1. Gold Standard Publication Checklist of study quality; Table S2. Alpha diversity of pure phenolic compounds and extracts.
