# Supplementary material for: Polyphenols as Prebiotics in the Management of High-Fat Diet-Induced Obesity: A Systematic Review of Animal Studies
Source: Foods. 2021 Feb 2;10(2):299. doi: 10.3390/foods10020299 (PMC7913110; doi:10.3390/foods10020299)
Supplement: Supplementary file 1 [file foods-10-00299-s001.zip › Supplementary/Supplementary F5.docx]

Figure S5: Effect of polyphenols on adiposity

|  |
| --- |
| *SL-Significantly Low, *NS-Not Significant, VAT-Visceral Adipose Tissue, SAT-Subcutaneous Adipose Tissue, TBF-Total Body Fat. ** compared to HFD*. Studies that tested more than one compound/dose: VAT (48, 53, 59), TBF (38) |
