# Supplementary material for: Polyphenols as Prebiotics in the Management of High-Fat Diet-Induced Obesity: A Systematic Review of Animal Studies
Source: Foods. 2021 Feb 2;10(2):299. doi: 10.3390/foods10020299 (PMC7913110; doi:10.3390/foods10020299)
Supplement: Supplementary file 1 [file foods-10-00299-s001.zip › Supplementary/Supplementary F6.docx]

Figure S6: Effect of polyphenols on lipid profile

|  |
| --- |
| *SL-Significantly Low, *SH-Significantly High, *NS-Not Significant, TC-Total cholesterol, TAG-Triacylglyceride, LDL-Low Density Lipoprotein, HDL-High Density Lipoprotein. **compared to HFD*. Studies that tested more than one compound/dose: TC (32, 46, 51, 54, 58, 59), TAG (38, 53, 58, 59, 62, 63, 67), LDL (46, 51, 63) (52, 60), HDL (32, 46, 48, 51, 59, 62) |
