# Supplementary material for: Polyphenols as Prebiotics in the Management of High-Fat Diet-Induced Obesity: A Systematic Review of Animal Studies
Source: Foods. 2021 Feb 2;10(2):299. doi: 10.3390/foods10020299 (PMC7913110; doi:10.3390/foods10020299)
Supplement: Supplementary file 1 [file foods-10-00299-s001.zip › Supplementary/Supplementary F7.docx]

Figure S7: Effect of polyphenols of glucose homeostasis

|  |
| --- |
| *SL-Significantly Low, *NS-Not Significant, FBG-Fasting Blood Glucose, GTT-Glucose tolerance Test, FI-Fasting Insulin, ITT-Insulin Tolerance Test, HOMA-IR- Homoeostasis Model Assessment–Estimated Insulin Resistance. **compared to HFD.* Studies that tested more than one compound/dose: FBG (46, 53, 57, 62, 64), GTT (37-39, 64, 65), FI (41, 53, 67), ITT (67), HOMA-IR (38, 41, 53, 62, 65) |
