# Supplementary material for: Polyphenols as Prebiotics in the Management of High-Fat Diet-Induced Obesity: A Systematic Review of Animal Studies
Source: Foods. 2021 Feb 2;10(2):299. doi: 10.3390/foods10020299 (PMC7913110; doi:10.3390/foods10020299)
Supplement: Supplementary file 1 [file foods-10-00299-s001.zip › Supplementary/Supplementary F8.docx]

Figure S8: Adipocytokines, C-Reactive Protein (CRP), and LPS/LBP

|  |
| --- |
| *SL-Significantly Low, *SH-Significantly High, *NS-Not Significant, TNFα- Tumour Necrosis Factor Alpha, IL-6-Interleukin -6, MCP-1- Monocyte Chemoattractant Protein-1, APN-Adiponectin. **compared to HFD.* Studies that tested more than one compound/dose: TNFα (45, 48), IL-6 (45, 58), MCP-1 (38, 42), LPS (38, 41, 45, 58, 67) |
