# Supplementary material for: Polyphenols as Prebiotics in the Management of High-Fat Diet-Induced Obesity: A Systematic Review of Animal Studies
Source: Foods. 2021 Feb 2;10(2):299. doi: 10.3390/foods10020299 (PMC7913110; doi:10.3390/foods10020299)
Supplement: Supplementary file 1 [file foods-10-00299-s001.zip › Supplementary/Supplementary F9.docx]

Figure S9: Beta-diversity of (a) pure phenolic compounds and (b) phenolic extracts

| a | b |
| --- | --- |
| ND-Normal Diet, HFD-High Fat Diet | |
