# Supplementary material for: Polyphenols as Prebiotics in the Management of High-Fat Diet-Induced Obesity: A Systematic Review of Animal Studies
Source: Foods. 2021 Feb 2;10(2):299. doi: 10.3390/foods10020299 (PMC7913110; doi:10.3390/foods10020299)
Supplement: Supplementary file 1 [file foods-10-00299-s001.zip › Supplementary/Supplementary T1.docx]

Table S1: Gold Standard Publication Checklist of study quality

|  | *Lopez et al., 18* | *Guo 2018 et al., (a)* | *Masumoto et al., 2016* | *Tung et al., 2016* | *Porras 2017* | *Brandt et al., 2018* | *Guo et al., 2018(b)* | *Cremonini et al., 2019* | *Campbell et al., 2019* | *Zheng et al., 2018* | *Xie et al., 2018* | *Sung et al., 2017* | *Liu et al.,2019* | *Ushiroda et al., 2019* | *Sheng et al., 2018* | *Liu et al., 2017* | *Wang et al.,2019* | *Lioa et al.,18* | *Zhao et al., 2017* | *Yang et al., 2019* |
| --- | --- | --- | --- | --- | --- | --- | --- | --- | --- | --- | --- | --- | --- | --- | --- | --- | --- | --- | --- | --- |
| *Research question specified and clear?* | 1 | 1 | 1 | 1 | 1 | 1 | 1 | 1 | 1 | 1 | 1 | *0* | 1 | 1 | 1 | 1 | 1 | 1 | 1 | 1 |
| *Method of allocation to treatment group: i.e. Animals randomized across groups?* | 1 | 0 | 1 | 1 | 0 | 1 | 1 | 0 | 1 | 0 | 1 | *1* | 0 | 0 | 1 | 0 | 0 | 1 | 1 | 1 |
| *Sample-size calculation before start of experiment* | 0 | 0 | 0 | 0 | 0 | 0 | 0 | 0 | 0 | 0 | 0 | *0* | 0 | 0 | 0 | 0 | 0 | 0 | 0 | 0 |
| *Concealment of allocation?* | 0 | 0 | 0 | 0 | 0 | 0 | 0 | 0 | 0 | 0 | 0 | *0* | 0 | 0 | 0 | 0 | 0 | 0 | 0 | 0 |
| *Group characteristics clearly described?** | 1 | 1 | 1 | 1 | 1 | 1 | 1 | 1 | 1 | 1 | 1 | *1* | 1 | 1 | 1 | 1 | 1 | 1 | 1 | 1 |
| *Correct control group used?* | 1 | 1 | 1 | 1 | 1 | 1 | 1 | 1 | 1 | 1 | 1 | *1* | 1 | 1 | 1 | 1 | 1 | 1 | 1 | 1 |
| *Number of animals per group clear?* | 1 | 1 | 1 | 1 | 1 | 0 | 1 | 1 | 1 | 1 | 1 | *1* | 1 | 1 | 0 | 1 | 1 | 1 | 1 | 1 |
| *Age, and sex of the animal* | 1 | 1 | 1 | 1 | 1 | 1 | 1 | 1 | 1 | 1 | 1 | *1* | 1 | 1 | 1 | 1 | 1 | 1 | 0 | 1 |
| *Weight of the animal* | 1 | 0 | 0 | 0 | 0 | 0 | 0 | 0 | 0 | 0 | 0 | *0* | 0 | 0 | 0 | 0 | 1 | 0 | 1 | 0 |
| *Complete outcome data?* | 1 | 1 | 1 | 1 | 1 | 1 | 1 | 1 | 1 | 1 | 1 | *1* | 1 | 1 | 1 | 1 | 1 | 1 | 1 | 1 |
| *Description of how the disease or intervention is defined in the animal* | 1 | 1 | 1 | 1 | 1 | 1 | 1 | 1 | 1 | 1 | 1 | *1* | 1 | 1 | 1 | 1 | 1 | 1 | 1 | 1 |
| *Time schedule (Day and time of intervention within experiment)* | 0 | 0 | 0 | 0 | 0 | 0 | 0 | 0 | 0 | 0 | 0 | *0* | 0 | 0 | 0 | 0 | 0 | 0 | 0 | 0 |
| *Dose and/or frequency of intervention* | 1 | 1 | 0 | 1 | 1 | 1 | 1 | 1 | 1 | 1 | 1 | *1* | 1 | 1 | 1 | 1 | 1 | 1 | 1 | 1 |
| *Method of sampling (blood, urine, etc.)* | 1 | 1 | 0 | 1 | 0 | 0 | 1 | 1 | 0 | 1 | 0 | *0* | 0 | 0 | 0 | 1 | 0 | 0 | 0 | 0 |
| *Time of sampling (blood, urine, etc.)* | 0 | 0 | 0 | 0 | 0 | 0 | 0 | 0 | 0 | 0 | 0 | *0* | 0 | 0 | 0 | 0 | 0 | 0 | 0 | 0 |
| *Excluded animals (numbers and reasons why they were excluded)* | 0 | 0 | 0 | 0 | 0 | 0 | 0 | 0 | 0 | 0 | 0 | *0* | 0 | 0 | 0 | 0 | 0 | 0 | 1 | 0 |
| *Total numbers of animals included in the statistical analyses* | 0 | 0 | 1 | 0 | 1 | 1 | 0 | 1 | 0 | 1 | 0 | *1* | 1 | 0 | 1 | 1 | 0 | 0 | 1 | 0 |
| *Description of compliance to national regulatory principles* | 1 | 1 | 1 | 1 | 1 | 1 | 1 | 1 | 1 | 1 | 1 | *1* | 1 | 1 | 1 | 1 | 1 | 1 | 1 | 1 |
| *statement of potential conflicts of interest* | 1 | 1 | 0 | 1 | 1 | 1 | 1 | 1 | 1 | 1 | 0 | *1* | 1 | 1 | 1 | 1 | 1 | 1 | 1 | 1 |
| *Total/19* | 13 | 11 | 10 | 12 | 11 | 11 | 12 | 12 | 11 | 12 | 10 | 11 | 11 | 10 | 11 | 12 | 11 | 11 | 13 | 11 |

|  | *Luo et al., 2019* | *Yong-Feng et al., 2019* | *Zhu et al., 18* | *Lee et al., 2019* | *Collins et al., 2016* | *Guo et al., 2019* | *Cheng et al., 2016* | *Liu et al., 2016* | *Griffin et al.,2017* | *Li et al., 2019* | *Ma et al.,2019* | *Van Hul et al., 2017* | *Anhe et al., 2015* | *Anhe et al., 2017* | *Anhe et al., 2018* | *Chen et al., 2018* | *Xu et al., 2019(a)* |
| --- | --- | --- | --- | --- | --- | --- | --- | --- | --- | --- | --- | --- | --- | --- | --- | --- | --- |
| *Research question specified and clear?* | 1 | 0 | 1 | 1 | 1 | 1 | 0 | 1 | 1 | 1 | 1 | 1 | 1 | 1 | 1 | 1 | 1 |
| *Method of allocation to treatment group: i.e. Animals randomized across groups?* | 1 | 1 | 1 | 0 | 1 | 1 | 1 | 1 | 1 | 0 | 1 | 1 | 1 | 0 | 1 | 1 | 1 |
| *Sample-size calculation before start of experiment* | 0 | 0 | 0 | 0 | 0 | 0 | 0 | 0 | 0 | 0 | 0 | 0 | 0 | 0 | 0 | 0 | 0 |
| *Concealment of allocation?* | 0 | 0 | 0 | 0 | 0 | 0 | 0 | 0 | 0 | 0 | 0 | 0 | 0 | 0 | 0 | 0 | 0 |
| *Group characteristics clearly described?** | 1 | 1 | 1 | 1 | 1 | 1 | 1 | 1 | 1 | 1 | 1 | 1 | 1 | 1 | 1 | 1 | 1 |
| *Correct control group used?* | 1 | 1 | 1 | 1 | 1 | 1 | 1 | 1 | 1 | 1 | 1 | 1 | 1 | 1 | 1 | 1 | 1 |
| *Number of animals per group clear?* | 1 | 1 | 1 | 0 | 1 | 1 | 1 | 1 | 1 | 1 | 1 | 1 | 1 | 1 | 1 | 1 | 1 |
| *Age, and sex of the animal* | 1 | 1 | 0 | 1 | 1 | 1 | 1 | 1 | 1 | 0 | 0 | 1 | 1 | 1 | 1 | 1 | 1 |
| *Weight of the animal* | 0 | 0 | 1 | 0 | 0 | 0 | 0 | 0 | 1 | 1 | 0 | 0 | 0 | 0 | 0 | 1 | 0 |
| *Complete outcome data?* | 1 | 1 | 1 | 1 | 1 | 1 | 1 | 1 | 0 | 1 | 1 | 1 | 1 | 0 | 1 | 1 | 1 |
| *Description of how the disease or intervention is defined in the animal* | 1 | 1 | 1 | 1 | 1 | 1 | 1 | 1 | 1 | 1 | 1 | 1 | 1 | 1 | 1 | 1 | 1 |
| *Time schedule (Day and time of intervention within experiment)* | 0 | 0 | 0 | 0 | 0 | 0 | 0 | 0 | 0 | 0 | 0 | 0 | 0 | 0 | 0 | 0 | 0 |
| *Dose and/or frequency of intervention* | 1 | 1 | 1 | 1 | 1 | 1 | 1 | 0 | 1 | 1 | 1 | 1 | 1 | 1 | 1 | 1 | 1 |
| *Method of sampling (blood, urine, etc.)* | 0 | 0 | 1 | 0 | 0 | 1 | 0 | 1 | 0 | 0 | 0 | 1 | 1 | 1 | 1 | 0 | 0 |
| *Time of sampling (blood, urine, etc.)* | 0 | 0 | 0 | 0 | 0 | 0 | 0 | 0 | 0 | 0 | 0 | 0 | 0 | 0 | 0 | 0 | 0 |
| *Excluded animals (numbers and reasons why they were excluded)* | 0 | 0 | 1 | 0 | 0 | 0 | 0 | 0 | 0 | 0 | 0 | 0 | 0 | 0 | 0 | 0 | 0 |
| *Total numbers of animals included in the statistical analyses* | 0 | 1 | 1 | 0 | 1 | 0 | 0 | 0 | 1 | 1 | 0 | 0 | 1 | 1 | 0 | 0 | 0 |
| *Description of compliance to national regulatory principles* | 1 | 1 | 1 | 1 | 1 | 1 | 1 | 1 | 1 | 1 | 1 | 1 | 1 | 1 | 1 | 1 | 1 |
| *statement of potential conflicts of interest* | 1 | 1 | 1 | 1 | 1 | 1 | 0 | 0 | 1 | 1 | 1 | 1 | 1 | 1 | 1 | 1 | 1 |
| *Total* | 11 | 11 | 14 | 9 | 12 | 12 | 9 | 10 | 12 | 11 | 10 | 12 | 13 | 11 | 12 | 12 | 11 |

|  | *Dey et al., 2019* | *Wu et al., 2018* | *Wu et al.,2019* | *Vezza et al.,2019* | *Henning et al., 18* | *Zhoa et al., 2019* | *Lee et al., 2018* | *Xu et al., 2019(b)* |
| --- | --- | --- | --- | --- | --- | --- | --- | --- |
| *Research question specified and clear?* | 1 | 1 | 1 | 1 | 1 | 1 | 1 | 1 |
| *Method of allocation to treatment group: i.e. Animals randomized across groups?* | 0 | 1 | 1 | 0 | 0 | 1 | 0 | 1 |
| *Sample-size calculation before start of experiment* | 0 | 0 | 0 | 0 | 0 | 0 | 0 | 0 |
| *Concealment of allocation?* | 0 | 0 | 0 | 0 | 0 | 0 | 0 | 0 |
| *Group characteristics clearly described?** | 1 | 1 | 1 | 1 | 1 | 1 | 1 | 1 |
| *Correct control group used?* | 1 | 1 | 1 | 1 | 1 | 1 | 1 | 1 |
| *Number of animals per group clear?* | 1 | 1 | 1 | 1 | 1 | 1 | 1 | 1 |
| *Age, and sex of the animal* | 1 | 1 | 1 | 1 | 1 | 0 | 0 | 1 |
| *Weight of the animal* | 0 | 0 | 1 | 0 | 1 | 1 | 1 | 0 |
| *Complete outcome data?* | 1 | 1 | 1 | 1 | 1 | 1 | 1 | 1 |
| *Description of how the disease or intervention is defined in the animal* | 1 | 1 | 1 | 1 | 1 | 1 | 1 | 1 |
| *Time schedule (Day and time of intervention within experiment)* | 0 | 0 | 0 | 0 | 0 | 0 | 0 | 0 |
| *Dose and/or frequency of intervention* | 1 | 1 | 1 | 1 | 1 | 1 | 1 | 1 |
| *Method of sampling (blood, urine, etc.)* | 1 | 0 | 0 | 0 | 0 | 0 | 1 | 0 |
| *Time of sampling (blood, urine, etc.)* | 0 | 0 | 0 | 0 | 0 | 0 | 0 | 0 |
| *Excluded animals (numbers and reasons why they were excluded)* | 1 | 0 | 1 | 0 | 0 | 0 | 0 | 0 |
| *Total numbers of animals included in the statistical analyses* | 1 | 0 | 1 | 1 | 1 | 1 | 1 | 0 |
| *Description of compliance to national regulatory principles* | 1 | 1 | 1 | 1 | 1 | 1 | 1 | 1 |
| *statement of potential conflicts of interest* | 0 | 1 | 1 | 1 | 1 | 1 | 1 | 1 |
| *Total* | 12 | 11 | 14 | 11 | 12 | 12 | 12 | 11 |
