# Supplementary material for: Polyphenols as Prebiotics in the Management of High-Fat Diet-Induced Obesity: A Systematic Review of Animal Studies
Source: Foods. 2021 Feb 2;10(2):299. doi: 10.3390/foods10020299 (PMC7913110; doi:10.3390/foods10020299)
Supplement: Supplementary file 1 [file foods-10-00299-s001.zip › Supplementary/Supplementary T2.docx]

Table S2: Alpha diversity of pure phenolic compounds and extracts

| Diversity indices | PPC | | | PE | | |
| --- | --- | --- | --- | --- | --- | --- |
|  | SH | SL | NS | SH | SL | NS |
| ACE | (28, 62) |  | (35) | (40) | (53) | (53) |
| Choa1 | (28, 36, 62) | (48) | (34, 35, 48, 61) | (30, 40, 56) |  | (37, 42, 55, 59) |
| OTU | (28) |  | (22, 35) | (38) |  | (38) |
| Shannon | (36, 50) | (32, 48, 49, 61) | (23, 28, 32, 35, 48) | (30, 52, 54, 56) | (53) | (37, 42, 53, 55, 59) |
| Simpson |  |  | (28, 31, 35, 60) |  | (54) | (59) |
| Inverse Simpson |  |  |  |  |  | (54) |
| PD |  |  |  |  |  | (42) |

*SH-Significantly High, *SL-Significantly Low, *NS-Not Significant, ACE-Abundance-Based Coverage Estimator, OTU-Operational Taxonomic Unit, PD- Phylogenetic Diversity. ** compared to HFD.*
