# Supplementary figures and images for: Polyphenols as Prebiotics in the Management of High-Fat Diet-Induced Obesity: A Systematic Review of Animal Studies
Source: Foods. 2021 Feb 2;10(2):299. doi: 10.3390/foods10020299 (PMC7913110; doi:10.3390/foods10020299)

Figure S1-Search strategy (PubMed)


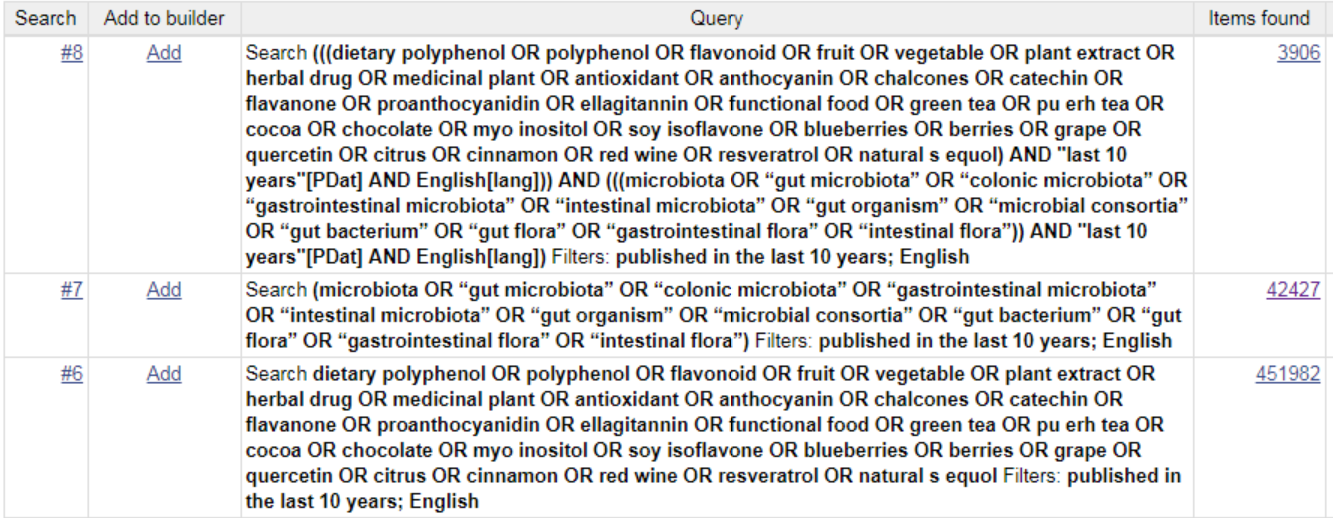

Supplement: Supplementary file 1 [file foods-10-00299-s001.zip › Supplementary/Supplementary F1.docx]
